# Microbial metabolic networks in a complex electrogenic biofilm recovered from a stimulus-induced metatranscriptomics approach

**DOI:** 10.1038/srep14840

**Published:** 2015-10-07

**Authors:** Shun’ichi Ishii, Shino Suzuki, Aaron Tenney, Trina M. Norden-Krichmar, Kenneth H. Nealson, Orianna Bretschger

**Affiliations:** 1 Department of Microbial and Environmental Genomics, J. Craig Venter Institute, La Jolla, CA 92037, USA; 2 R&D Center for Submarine Resources, Japan Agency for Marine-Earth Science and Technology (JAMSTEC), Nankoku, Kochi 783-8502; 3 Kochi Institute for Core Sample Research, JAMSTEC, Nankoku, Kochi 783-8502; 4 Project Team for Development of New-generation Research Protocol for Submarine Resources, JAMSTEC, Nankoku, Kochi 783-8502; 5 Department of Earth Sciences, University of Southern California, Los Angeles, CA 90089, USA

## Abstract

Microorganisms almost always exist as mixed communities in nature. While the significance of microbial community activities is well appreciated, a thorough understanding about how microbial communities respond to environmental perturbations has not yet been achieved. Here we have used a combination of metagenomic, genome binning, and stimulus-induced metatranscriptomic approaches to estimate the metabolic network and stimuli-induced metabolic switches existing in a complex microbial biofilm that was producing electrical current via extracellular electron transfer (EET) to a solid electrode surface. Two stimuli were employed: to increase EET and to stop EET. An analysis of cell activity marker genes after stimuli exposure revealed that only two strains within eleven binned genomes had strong transcriptional responses to increased EET rates, with one responding positively and the other responding negatively. Potential metabolic switches between eleven dominant members were mainly observed for acetate, hydrogen, and ethanol metabolisms. These results have enabled the estimation of a multi-species metabolic network and the associated short-term responses to EET stimuli that induce changes to metabolic flow and cooperative or competitive microbial interactions. This systematic meta-omics approach represents a next step towards understanding complex microbial roles within a community and how community members respond to specific environmental stimuli.

Microbial community activities define the rates of key biogeochemical cycles across the globe and are important to biotechnology, bioremediation, industrial and clinical applications^1^. While the importance of microbial community activities is widely recognized, it is challenging to acquire details about the specific microbial interaction networks that enable community functions^2^. Understanding these microbial networks is essential to expanding our predictive capability of the factors that control community function, adaptation and evolution^3^. Inherent complexities associated with understanding microbial networks include specifying taxonomic composition, genetic potential, metabolic activity^1^,^4^,^5^, and also functional adaptability of each community member to environmental perturbations and how stimuli affect community function as a whole^6^,^7^. Additionally, microbial interaction networks must accurately describe the cooperative, competitive, or neutral interactions that may occur between microbes^8^,^9^. To-date microbial metabolic interactions have been explored using flux analyses between defined co-cultures^10^ and tri-cultures^11^ of microbial isolates under defined conditions, or via community reconstruction using five isolated dominant microbes from a more complex consortium^12^. However, these types of approaches are not practical for highly diverse mixed communities and do not address the specific genetic responses induced as a function of a given environmental stimulus.

Several groups have begun investigating and describing microbial networks in highly diverse communities relative to taxonomic composition, genetic potential and metabolic activity. Cultivation-independent molecular surveys based on conserved marker genes (such as the 16S rRNA gene) have provided a greater understanding of community taxonomic compositions and co-occurrence patterns^8^. DNA-based metagenomic analyses have more precisely defined both the taxonomic compositions and collective gene pools of many highly complex microbial communities, providing greater insights into the metabolic potentials of whole communities^13^. Recently, high-quality microbial draft genomes of community members have been successfully recovered from deeply sequenced metagenomes^14^,^15^, which elevates the level of resolution from a whole community to individual members. However, such DNA-based studies cannot address actual microbial activities.

Metatranscriptomic mRNA-based analyses are now used to quantify transcripts within complex microbial communities in many different environments^16^,^17^,^18^, thus enabling the characterization of gene activity within entire communities directly through measuring levels of gene expression. However, many of these studies faced challenges relative to correlating gene activities with specific environmental variables because multiple variables (e.g., temperature, light, and redox) often change simultaneously. In addition, the genetic background can shift temporally^19^ and/or spatially^20^ along with community composition changes, adding yet another challenge to the interpretation of metatranscriptomic data. While these data sets have contributed significant new knowledge relative to describing whole community activities, they cannot specifically address each member’s functional role, metabolic interactions, or adaptability to environmental perturbations.

To address these challenges we have developed an experimental strategy called “stimulus-induced metatranscriptomics”^21^. The strategy enables the characterization of transcriptional responses to specific environmental changes by applying focused stimuli and analyzing gene expression profiles before the community taxonomic composition changes under the new environmental condition. By combining genome binning strategies, we are able to describe metabolic activity and functional adaptability at both a community- and strain-level resolution. In our previous study, we applied this multi-pronged strategy to identify functional microbes and genes associated with extracellular electron transfer (EET)^21^.

EET-mediated reactions are widespread in subsurface environments where iron- and manganese-oxide reduction drives the anaerobic oxidation of organic matter^22^. We utilized bioelectrochemical systems to enrich a functional multi-species biofilm (over 100 species) from wastewater^23^, and identified two specific EET-active microbes, and their gene cassettes, that rapidly responded to changes in electrode surface potentials^21^. The previous study focused on describing the competitive respiratory reactions including solid surface reduction via EET, and identifying key EET-active members. However, it was still unclear how the two EET-active microbes were metabolically interacting with each other, or with other community members in terms of cooperative (*e.g*. species one produces a metabolite that can be consumed by species two) and competitive (*e.g*. species one and two both require the same metabolite) relationships. Electron donors for the EET-active microbes were supplied via the decomposition of complex organic matter in wastewater, which is usually performed by other microbes^24^; thereby, establishing successful microbial metabolic networks is necessary for maintaining a functional EET-active community.

Here we address how the EET-active microbes metabolically interact with each other and with other microbial members using a combination of improved genome binning strategies and an optimized stimulus-induced metatranscriptomics analytical approach. By improving the draft genome binning process, we increased the number of high-coverage Bin-genomes from four to eleven, enabling a more comprehensive analysis of the metabolic connections within the community. We further expanded the metatranscriptomic analytical scheme to include marker gene sets for cell activities to identify positive or negative response to the given stimulus, and for metabolic activities to identify specific metabolism changes. This significantly improved omics-based analytical strategy now offers a robust approach for estimating microbial interaction networks and understanding microbial community adaptation strategies.

## Results

### Analytical scheme overview

**Figure 1:**
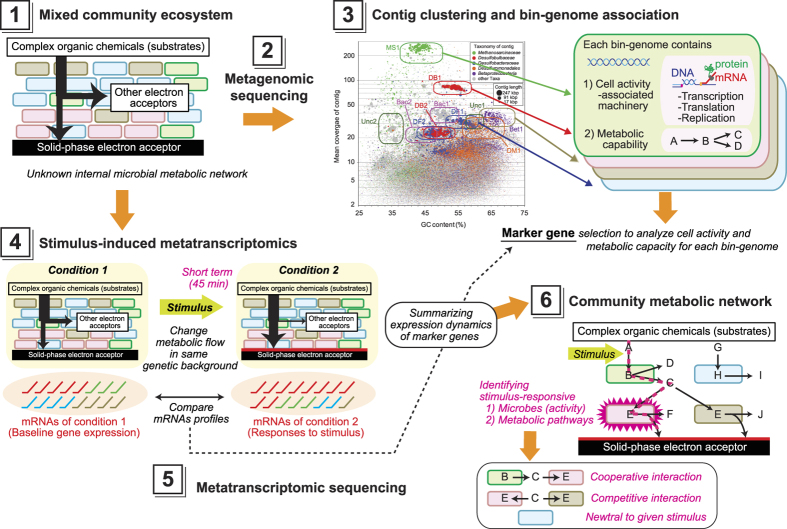
Scheme for the analytical approach used to describe the microbial networks in a complex EET-active community. (Step **1**) Enrichment of an EET-active microbial ecosystem; (Step **2**) Metagenomic sequencing and analysis of the community; (Step **3**) Bin-genome association by contig clustering. Each cluster indicates a Bin-genome of a community member, which includes coding sequences for cell activity and available metabolic pathways; (Step **4**) stimulus-induced metatranscriptomics involving the application of a specific EET-condition via stimulus addition and biofilm sampling with DNA and mRNA extraction; (Step **5**) Metatranscriptomic sequencing and subsequent comparative analyses of gene expression profiles of the whole community and each community member, which are executed via marker gene sets correlated with important “cell activity” and “metabolic” functions; (Step **6**) Construction of the community metabolic network for the dominant microbes within the community.

Figure 1 summarizes our newly-developed approach for describing metabolic networks within the complex EET-active community. This approach addresses microbial activity as well as cooperative, competitive, or neutral metabolic interactions between dominant microbes within the community as described in Fig. 1 step 6.

### Bin-genome association for highly abundant strains

An EET-active electrogenic biofilm was established in a MFC bio-reactor repeatedly fed with wastewater for over 2 years^23^,^25^. The wastewater contained variable organic compounds that could be used for microbial fermentation and various microbial respiration processes with soluble electron acceptors and the solid electrode via EET. Our first metagenomic analysis of this community recovered four Bin-genomes and one pan-genome of the dominant strains^21^; however, more Bin-genomes were needed to obtain comprehensive interspecies metabolic networks.

The recovery of additional Bin-genomes was accomplished by an improved sequence assembly, which yielded 169,740 contigs (Supplementary Table S1). We clustered the contigs by using mean contig coverage vs. G+C content plots, which showed clustering when the mean coverage was >20 (Supplementary Fig. S2). Then, we refined the clusters by using differential coverage binning method and their tetra-nucleotide frequencies^15^. From the contig clusters, we recovered thirteen Bin-genomes overall (Table 1), two of which were a mixture of genomes from at least two different microbes (Supplementary Table S2–S4), and six of which were high-quality draft genomes as assessed by the HMP criteria (Supplementary Table S5)^26^. The genome mixtures were omitted from further analyses. From the Bin-genome clustering, we successfully identified the Bin-genome of the most dominant *Desulfuromonadaceae* strain DM1 from *Desulfuromonadales* pan-genome DM of the previous study^21^, which is important for analyzing the strain-based metabolic network. In total, eleven Bin-genomes were utilized for reconstructing the metabolic networks within the community. A detailed description and discussion of these results can be found in Supporting Information (Supplementary Discussion, Supplementary Fig. S3–S7, and Table S3–S6).

### Bin-genome frequency within the community

**Figure 2:**
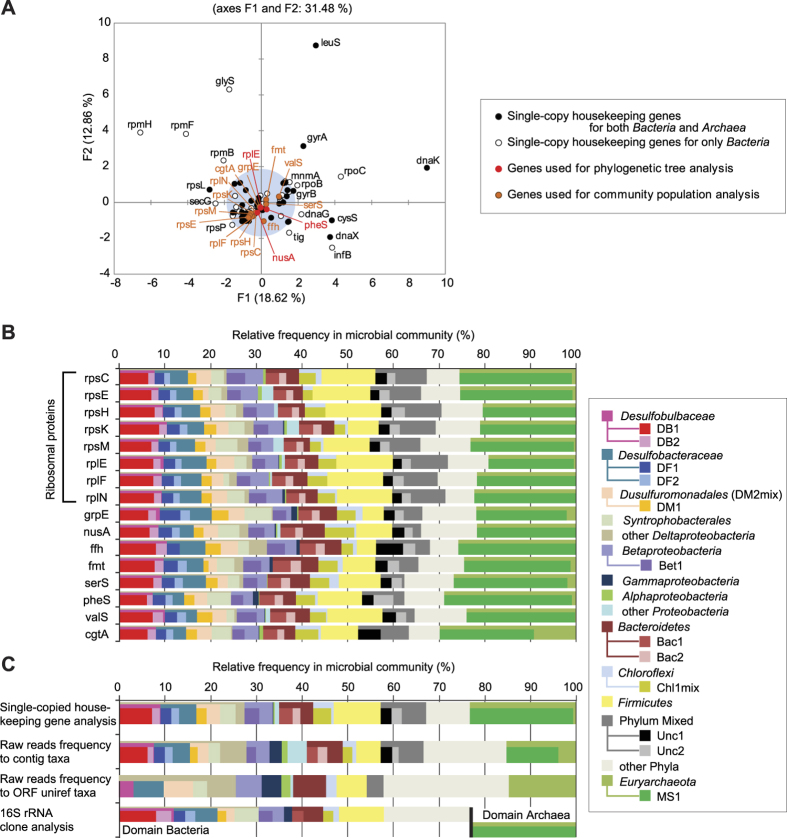
Single-copy housekeeping gene-based analysis to profile microbial diversity using metagenomic analyses and Bin-genome clustering. (Panel **A**) shows principal component analysis (PCA) diagram for 107 single-copy bacterial housekeeping genes based on existence in each Bin-genome (Supplementary Table S3). Genes determined as suitable for the community analyses are clustered within gray area. Names of sixteen core-genes used for microbial community population analysis are described in red/orange colors. (Panel **B**) shows the taxonomic composition of the microbial community based on core-gene frequencies of each taxon and Bin-genome (bars inside). (Panel **C**) shows comparison of microbial community compositions between three different methods of metagenomic analyses and 16S rRNA clone analyses separately conducted for domains *Bacteria* and *Archaea*.

Bin-genome frequencies within the community are essential for normalizing and calculating mRNA/DNA ratios, which is the quantitative value used to determine gene expression levels. Metagenomics-based community composition analysis was performed by using prokaryotic single copy housekeeping genes (Fig. 2). Within 107 reported housekeeping genes^21^, we selected sixteen core genes that are present in all eleven Bin-genomes (Fig. 2A). The relative frequencies of each Bin-genome were determined based on coverage of the selected core genes for each Bin-genome (Fig. 2B); then the community composition was compared using different methods (Fig. 2C). The results showed that 52% of the microbial population was occupied by the eleven dominant Bin-genomes (Table 1). This approach revealed a more accurate view of community composition relative to other methods including 16S rRNA gene-based clone analysis^21^ and raw read frequencies of each taxon (Supplementary Table S6–S7). The detailed discussion for the improved community composition analysis can be found in Supplementary Discussion.

### Gene expression trends during different EET conditions

In our previous study, we identified EET-responsive microbes (Bin-genome DB1 and Pan-genome DM) and genes within the electrode-associated EET-active biofilm by the dynamic analyses of gene-expression profiles between three sequential operational condition changes within 3-hr time period: 1) baseline condition related to static EET-activity (MFC) at 5 hours after wastewater replacement and stabilization of current generation with 70 mA/m^2^ of anodic current density; 2) increased EET rate with 430 mA/m^2^ of current density that was achieved by set-potential (SP) condition that held the anode surface potential as +100 mV vs SHE under potentiostatic operation; and 3) zero EET activity with no current generation achieved by open-circuit (OC) condition (Table 2)^24^. Stimuli were applied for 45 minutes until current production or open circuit potential was observed to stabilize for each operational change, then DNA and mRNA of the anodic biofilms were co-extracted and sequenced as described in previous study^24^. With these stimuli, the rector coulombic efficiencies and wastewater treatment times were also changed. Coulombic efficiencies were 23% for MFC, 58% for SP, and 0% for OC; and the wastewater treatment times were 10.0 day for MFC, 4.8 day for SP, and 18.3 day for OC, which were evaluated by long term operations^23^.

As a next step toward fully describing the microbial relationships within the electrogenic community including those two EET-responsive microbes, the present study utilized the same mRNA sequence datasets (Table 2), but applied different analyses with the goal of describing a comprehensive metabolic network. We analyzed the overall gene expression dynamics for each Bin-genome, comparative expression levels of coding sequences (CDSs) between two operational conditions by scatter plotting, and the top 10 highly-expressed CDSs among the conditions (Supplementary Fig. S7–S11 and Table S8–S14). These basic analyses were sufficient to confirm that the main EET-responsive strains in the community were strains DB1 and DM1 as reported previously^21^. However, the overview analyses could not determine whether these two strains were positively activated or stressed; or the nature of their metabolic relationships to other dominant strains in the community. To achieve a more detailed view of community systems biology, we compiled a new set of marker genes for describing microbial cell activities (Supplementary Data 1) and metabolic activities (Supplementary Data 2) from full lists of KEGG module (Supplementary Data 3) and KEGG orthology (Supplementary Data 4), and analyzed these gene sets independently for each of the eleven Bin-genomes.

### Microbial cell activity dynamics

**Figure 3:**
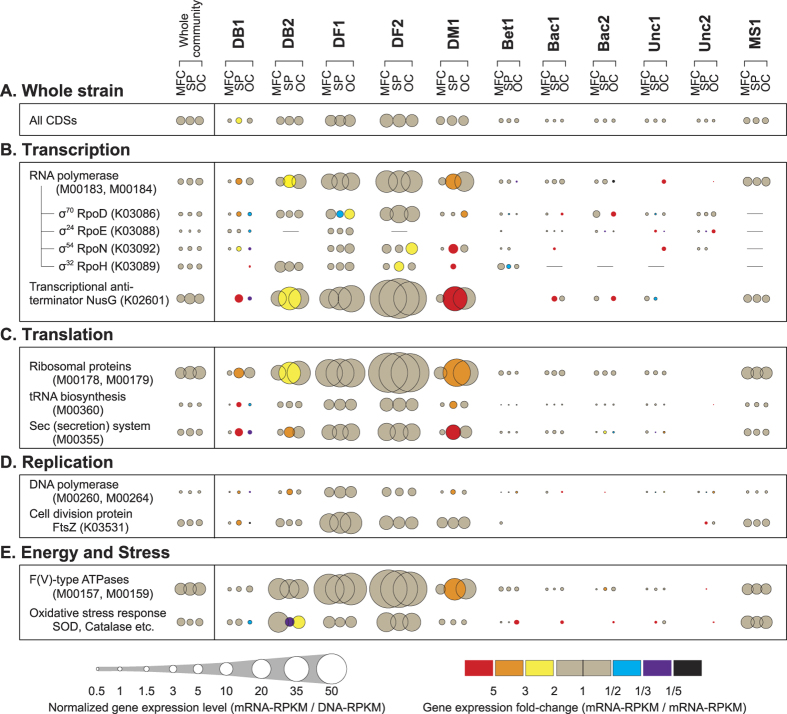
Overall gene expression levels and dynamics related to microbial cell activities for each Bin-genome. Mean gene expression levels were calculated from all CDSs in each Bin-genome (**A**). Gene expression levels and changes for selected marker gene families related to transcription (**B**), translation (**C**), replication (**D**), and energy and stress (**E**) were calculated (see Supplementary Data 1). Normalized gene expression levels (mRNA-RPKM/DNA-RPKM) for each Bin-genome under the three operational conditions is described by the size of circle, while gene expression dynamics (mRNA-RPKM/mRNA-RPKM) is described by the circle color of SP (expression fold-change from MFC to SP) and the circle color of OC (expression fold-change from SP to OC).

To compare the gene expression trends of cell activity-associated marker genes between the dominant strains, gene expression levels (mRNA-RPKM) were normalized by the DNA frequencies (DNA-RPKM), and both gene expression levels and dynamic changes were co-visualized in Fig. 3. The selected marker genes encode proteins that are ubiquitously essential for all life, including transcription, translation, and replication (Fig. 3B–D). In addition, genes encoding ATP synthase, superoxide reductase, and catalase were also employed as indicators for energy synthesis or stress responses, respectively (Fig. 3E). By combining them, we will enable to discuss about microbial cell activity dynamics for each strain within the given community.

Except for strain DB2 and the EET-responsive strains DB1 and DM1, the gene expression dynamics of the marker genes (Fig. 3B–E) were similar to those of the average/median for all CDSs in each Bin-genome (Fig. 3A and Supplementary Fig. S7). The gene expression levels and dynamics indicate that strains DF1, DF2 and DB2, which are likely sulfate reducers estimated from their phylogenetic position, were active members under all conditions but not EET-responsive. On the other hand, strains Bet1, Bac1, Bac2, Unc1, and Unc2 showed significantly low gene expression levels, indicating relatively low activity within the community.

More significant gene expression fold-changes were observed in EET-responsive strains DB1 and DM1 (Fig. 3). Especially, the gene encoding RpoH (σ^32^), which is a known stress response protein^27^, showed completely different expression trends between strains DB1 and DM1 (Fig. 3B). The *rpoH* gene of strain DB1 was highly expressed only under the OC condition, while that of strain DM1 was highly expressed under the SP condition. In addition, expression of the gene encoding cell division protein FtsZ^28^ was up-regulated under the SP condition and down-regulated under the OC condition in strain DB1; however, the *ftsZ* gene was consistently expressed in strain DM1 (Fig. 3D). These differential responses suggest that the EET-accelerated SP condition was a preferable situation for strain DB1; contrarily, the OC condition was a stressful condition, which are expected trends for an EET-active microbe. However, the other EET-responsive strain DM1 showed a more complicated trend, which suggest that the EET-accelerated SP condition was stressful due to a highly oxidative surface potential for the strain, or pH drop by H^+^ accumulation in the biofilm as a counter reaction of increased EET to the electrode. Subsequently, the strain DM1 adapted to the new environmental condition, as indicated by the modification of its gene expression profiles related to transcription and translation, which was not predicted from the previous research^21^. These results indicate that the EET-responsive strain DM1 may have been forced to reconstruct electron transfer pathways after sensing a change in electron acceptor availability.

The strain DB2 also showed over 2-fold higher gene expression for genes related to transcription, translation and replication as a result of the SP stimulus. Lower expression of oxidative stress response genes was also observed (Fig. 3E), which indicates that the strain DB2 may have been directly correlated with the EET reaction in a positive way.

### Microbial metabolic activity

**Figure 4:**
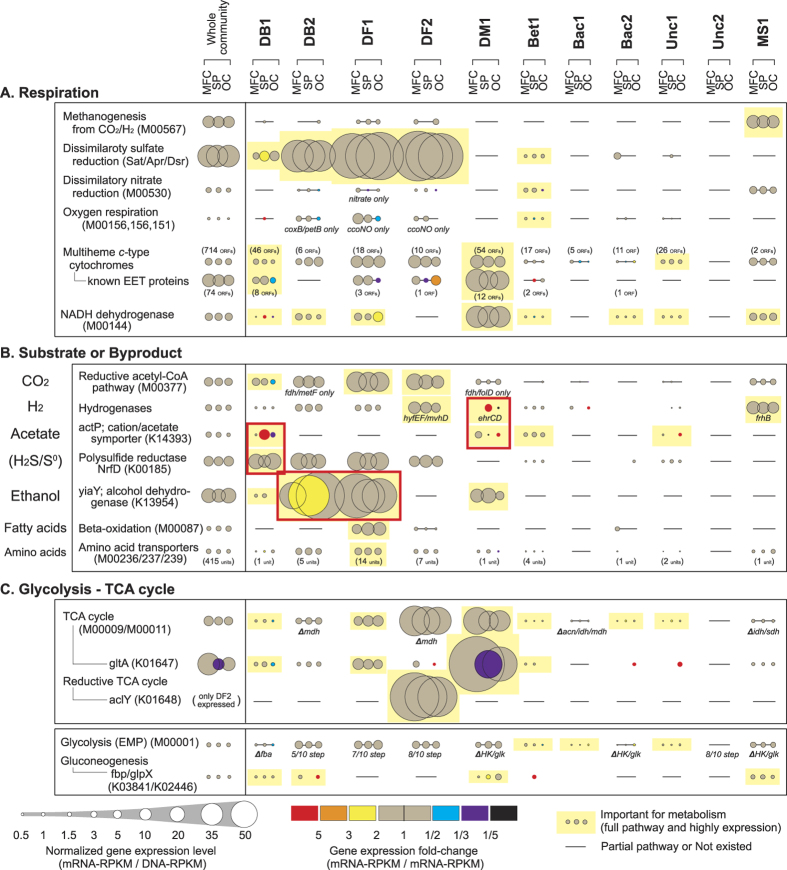
Overall gene expression levels and dynamics related to microbial metabolisms for each Bin-genome. Gene expression levels and changes were calculated (see Supplementary Data 2) for selected metabolism-related marker gene families associated to respiration (**A**) substrate consumption or byproduct production (**B**) and glycolysis and TCA cycle (**C**). Normalized gene expression levels (mRNA-RPKM/DNA-RPKM) for each Bin-genome under the three operational conditions is described by the size of circle, while gene expression dynamics (mRNA-RPKM/mRNA-RPKM) is described by the circle color of SP (expression fold-change from MFC to SP) and the circle color of OC (expression fold-change from SP to OC). Important gene sets for the metabolic pathway analyses within the community are indicated by yellow rectangles highlighting the circles. Bars indicate partial pathways of a given KEGG module or no existence of the KEGG orthology/module, and the elements of the incompleteness are described below the bar. Red rectangles indicate potential metabolism switches after stimuli addition.

Next, we analyzed the specific metabolic activities of each strain. Since a majority of metabolic interactions among microbes are related to fermentation and/or electron transfer reactions^24^, we analyzed the expression profiles of gene families associated with KEGG pathways or KEGG modules^29^ that are known to relate to these metabolic activities (Supplementary Data 2). From the list, we depicted gene expression trends of appropriate gene family modules for describing respiration pathways (Fig. 4A) ubiquitously occurring in the earth environments^30^, substrate consumption or byproduct production (Fig. 4B) which are well-known intermediate compounds within anaerobic metabolic networks^31^, and glycolysis and the TCA cycle (Fig. 4C) from highly expressed KEGG modules (Supplementary Data 3). Several marker genes were selected based on known key enzymes of the specific pathway (*e.g*. citrate synthase, *gltA* in TCA cycle) or highly expressed genes within the Bin-genomes (Supplementary Data 4) that correlated to a specific metabolic function (*e.g*. alcohol dehydrogenase, *yiaY* and cation/acetate symporter, *actP*).

### Metabolism for respiration

Gene expression levels of the five most ubiquitous respiration metabolisms^30^ indicated that methanogenesis, sulfate reduction, and EET to solid surfaces were actively occurring simultaneously in the community (Fig. 4A). The gene expression levels of each marker gene indicated that strain MS1 conducted hydrogenotrophic methanogenesis, while strains DB1, DB2, DF1, and DF2 conducted dissimilatory sulfate reduction; however, expression of sulfate reduction and methanogenesis related genes were not changed significantly among the three conditions, except for EET-active strain DB1. These results suggest that competitive respiration activities, relative to electrode respiration, were not immediately affected by changes in EET rates for most of the dominant strains in the community.

Dissimilatory metal reduction and electrode respiration via EET are known to be mediated by multi-heme *c*-type cytochromes (MH-cytCs)^32^. The EET-responsive strains DB1 and DM1 contained many genes encoding MH-cytCs, but their gene expression levels were not significantly changed under the three different EET conditions (Fig. 4A). We further analyzed twelve gene families that are known to encode key MH-cytCs for EET processes in model organisms *Geobacter* and *Shewanella*^33^,^34^, and found that the two strains include many of those key MH-cytCs. The key MH-cytCs of the two strains were highly expressed compared to all other MH-cytCs (Fig. 4A) and all CDSs (Fig. 3A), but not drastically changed by the EET stimuli. In contrast, the gene expression levels of several specific MH-cytC families were significantly changed by the EET stimuli in the two strains (Supplementary Data 2), suggesting that further characterization of different types of MH-cytCs may help to understand their roles in specific EET activities.

Only strain Bet1 showed the possible capability for nitrate and oxygen respiration, suggesting that the strain was an oxygen scavenger and/or dissimilatory nitrate reducer (Fig. 4A); however, the gene expression levels were relatively low, indicating that high concentrations of oxygen did not reach the anodic biofilm and the majority of the activity was associated with anaerobic respirations.

### Metabolism for electron donors

Understanding strain specific substrate utilization, and how substrates may be shared between strains, is extremely important for describing a comprehensive metabolic network. High molecular-weight complex organic chemicals in wastewater are diverse and have variable concentrations; therefore, the primary decomposition of complex organics may be conducted by diverse rare populations with specific metabolic niches. The rare population microbes in the biofilm did not yield high quality Bin-genomes because of low coverage (<20) in the metagenome (Supplementary Fig. S2). Thus, in this study, we focused our analyses on the more dominant microbial populations that appeared to metabolize the smaller compounds (sugars, amino acids, and fatty acids) that could be closely associated to terminal electron accepting reactions including EET. Complex substrate utilization generally happens by a combination of fermentative and respiratory reactions, where the fermentation byproducts are consumed as substrates for the respiratory microbes via interspecies metabolite transfer processes^24^. Figure 4B shows expression profiles of the selected highly expressed gene families/modules associated with substrate utilization during respiration processes or byproduct production during fermentation processes (the full list is shown in Supplementary Data 2). The results imply that hydrogen was consistently utilized by strains DF2 (as a chemolithoautotrophic sulfate reducer) and MS1 (as a hydrogenotrophic methanogen), while DF1 primarily consumed fatty acids and amino acids. Interestingly, strains DB1 and DM1 appeared to be competing for acetate utilization, while strains DF1 and DB2 were in competition for ethanol utilization under different EET conditions.

The gene expression profiles of the *actP* gene encoding the acetate uptake protein ActP^35^ indicated that strain DM1 was primarily responsible for acetate consumption under the MFC condition. However, strain DM1 showed a significantly lower *actP* expression under the SP condition, while strain DB1 had a 5-fold increase in *actP* expression relative to the MFC condition. These data indicate a competitive relationship between strains DB1 and DM1 with respect to acetate consumption, which is driven by EET rates. Further evidence for acetate utilization competition was provided by an analysis of the key genes related to central metabolic pathways including the *gltA* gene (encoding a key enzyme of the TCA cycle)^36^ and hydrogenase genes. The expression of the *gltA* gene was significantly decreased along with the *actP* gene in strain DM1 under the SP condition (Fig. 4C); however, expression of the hydrogenase genes was significantly increased (Fig. 4B). Specifically, the *ehrCD* genes encoding Ech hydrogenase complex (HyfEF) were significantly up-regulated for the strain DM1 under the SP condition (Supplementary Data 2). The Ech hydrogenase complex has been characterized as necessary for producing a reduced form of ferredoxin via hydrogen oxidation^37^. These trends suggest that strain DM1 stopped utilizing acetate and switched to hydrogen as its preferred electron donor during the EET-enhanced SP condition, which might be regulated by the stress-responsive sigma factor (RpoH) in strain DM1 (Fig. 3B). Interestingly, the SP-stimulus responsive hydrogenase EhrCD in strain DM1 is a different group from known respiratory uptake hydrogenase (HydB) in a model *Deltaproteobacterial* EET-active microbe, *Geobacter sulfurreducens*^38^. In addition, we recently reported that genus *Desulfuromonas* microbes were more associated with electro-negative surface potentials and acetate utilization as compared to genus *Geobacter* microbes^39^. Thus, we postulate that the *Desulfuromonadaceae* strain DM1 may have a mechanism to ‘sense’ surface potentials and adapt accordingly to rapidly reconstruct its protein profiles for both central carbon metabolism and EET-associated MH-cytCs.

The gene expression trends for the *yiaY* gene encoding alcohol dehydrogenase, which was the most highly expressed gene among alcohol dehydrogenases, also indicated a competitive relationship for ethanol utilization between two active sulfate reducers, strains DB2 and DF1 (Fig. 4B). Under the SP condition, strain DB2 appeared to be the dominant ethanol consumer and strain DF1 likely shifted from primarily ethanol to fatty acid consumption. The activation of strain DB2 under the SP condition was also supported by cell activity marker gene trends (Fig. 3). These trends indicate that strain DB2 was EET-responsive and preferred the SP condition, even though the strain seems not to correlate directly with EET-respiration from our knowledge to-date.

Under the MFC condition, strain DB1 showed high gene expression levels for the *nrfD* gene encoding a polysulfide reductase in their top 10 gene expression list (Supplementary Table S9); and interestingly, the *nrfD* gene expression trends were completely opposite to the acetate utilization-associated marker genes (Fig. 4B). The NrfD protein was originally identified as a conduit in the transfer of electrons from the quinone pool to terminal electron transfer components^40^; however, the protein has also been reported as an enzyme involved with the transformation of elemental sulfur to sulfide^41^. Since the preferred electron donor for strain DB1 under the MFC condition is unknown, the gene expression trends suggest that NrfD proteins might be utilized for electron donating under MFC condition, which might extract electrons from sulfide or elemental sulfur produced by active sulfate reducers.

### Metabolism for fermentation

Since highly variable organic matter exists in wastewater, a wide variety of fermentative strains could be maintained within the electrogenic community. An analysis of the gene expression levels of glycolysis-associated gene families indicated that strains Bet1, Bac1, and Unc1 may be sugar fermenters (Fig. 4C). However, their gene expression levels were too low to indicate any possible dynamic gene expression trends as a function of EET stimuli.

The contig clustering analysis showed many *Firmicutes*-assigned contigs, which are also potential fermenters, in the low mean coverage region (<20) that represents less than 1.5% relative frequency in the community (Fig. 2B, Supplementary Fig. S2, Table S7). However, in order to address such diverse but less dominant microbial metabolic activities, we will need to recover higher quality Bin-genomes of the fermenters via more sequencing.

### Describing microbial metabolic networks

**Figure 5:**
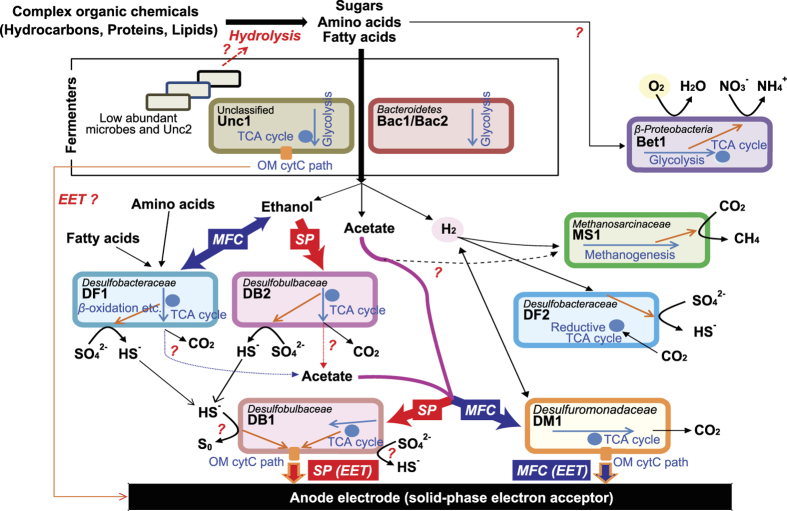
Estimated metabolic network between dominant microbes within the EET-active microbial community. Metabolic roles of eleven Bin-genomes (colored rounded rectangles) are estimated from the cell activity and metabolism-associated gene expression dynamics related to EET stimuli additions. Metabolism switches between MFC and SP conditions are described by thick arrows with blue (MFC) or red (SP) color. Intracellular and extracellular EET processes are described by orange arrows. Cytoplasmic carbon metabolic flows are described by right blue arrows.

From the combination of metagenomic, genome binning and stimulus-induced metatranscriptomics analyses, it is possible to hypothesize the metabolic networks that exist between the eleven dominant strains within the complex EET-active microbial community (Fig. 5). Since we added EET stimuli to the community, the suggested metabolic network and metabolism shifts between microbes were identified relative to the terminal electron accepting reaction to the anode electrode. EET-active strains DB1 and DM1 competed with each other for acetate utilization and the EET rate defined which strain prevailed, which was not expected from the previous study^21^. Strain DB1 was more active under the higher EET-rate condition (SP), while strain DM1 was the active acetate utilizer under the lower EET-rate condition (MFC). Strain DB1 also appeared to be correlated with sulfur metabolisms like dissimilatory sulfate reduction and sulfide oxidation, but the mechanisms that strain DB1 may use for these processes are not yet clear.

Another obvious metabolic shift observed under the SP condition was that primary ethanol consumption moved from strain DF1 to strain DB2 (Fig. 5). The upstream processes of wastewater treatment for converting complex organic compounds to volatile fatty acids, amino acids, alcohols, and H_2_ via microbial hydrolysis and fermentation reactions are still unknown from this EET stimulus-induced metatranscriptomic analyses. To achieve a greater understanding about those hydrolysis and fermentation processes, we hypothesize that specific fermentative substrates can be used as stimuli to produce new information about the processes via stimulus-induced metatranscriptomics methods.

### Discussion

To date, metabolic interaction networks within complex microbial communities have primarily been inferred from community dynamics^8^ and metagenomics^3^,^42^, and are now improving with the application of metatranscriptomic^43^,^44^ and metaproteomic^45^ approaches. Here we have significantly enhanced the current state-of-the-art by optimizing methods for extracting Bin-genomes and analyzing novel stimulus-induced metatranscriptomics data with appropriate marker gene sets (Fig. 1). The gene expression dynamics of cell activity-associated marker genes after the stimuli addition enabled classification of microbial responses to the specific stimulus as either positive, negative, or neutral (Fig. 3). The gene expression dynamics of metabolism-associated marker genes after the stimuli addition (Fig. 4) enabled the reconstruction of a metabolic pathway network and their pathway shifts by the given stimulus for dominant strains, and identified active metabolic pathways in the system (Fig. 5). Finally, we have successfully described the cooperative and competitive microbial interactions in the system, and identified how these relationships change as a function of induced stimuli, which has not yet been addressed by other types of studies.

From two EET-active strains, *Desulfobulbaceae* strain DB1 was more active under the SP condition than the MFC condition, while *Desulfuromonadaceae* strain DM1 showed an opposite trend. These same trends have been confirmed by longer-term microbial community population studies exploring continuous MFC and SP operation (Supplementary Fig. S12) that showed a relatively abundant population of family *Desulfobulbaceae* including strain DB1 in the SP enriched condition, while family *Desulfuromonadaceae* including strain DM1 was found as the most relatively abundant strain in the MFC condition^23^. These studies indicate that the gene expression trends of both cell activity and microbial metabolism observed in the short-term stimulus-induced metatranscriptomics are tightly correlated with longer-term microbial community dynamics. This consistency provides more evidence and stronger confidence in our estimated metabolic pathway networks and the metabolic switches predicted from our stimulus-induced metatranscriptomics approach. In addition, we operated triplicate MFCs fed with the same wastewater and conducted 16S rRNA community analyses throughout the operation. The results showed that all three reactors contained both *Desulfobulbaceae* and *Desulfuromonadaceae* phylotypes as abundant members in the anodic biofilms (Supplementary Fig. S13). These results also validate the observations yielded from the stimulus-induced metatranscriptomic experiments in this study. This level of understanding has not yet been achieved by other types of metagenomic, metatranscriptomic or metaproteomic studies.

The stimulus-induced metatranscriptomics analyses also indicate that two dominant *Desulfobulbaceae* strains, DB1 and DB2, were both more active under the SP condition (Figs 3 and 4), which suggests that the family *Desulfobulbaceae* may preferentially associate with more electropositive surface redox conditions as compared to other *Deltaproteobacterial* families in the electrogenic community. Family *Desulfobulbaceae* microbes have been reported in relative abundance in electrogenic biofilms that have been extracted from anodes in a sediment MFC^46^,^47^ and in a rice straw hydrolysate-fed MFC^48^, both of which contained sulfate in the reactors or sediments. Further, a filamentous *Desulfobulbaceae* strain has recently been identified as an important group in marine sediments for long distance EET processes from the sulfate-reducing zone to the oxygen-respiring zone^49^. These reports suggest that *Desulfobulbaceae* strains could be more competitive in sulfate-containing and EET-active sediments than *Geobacter* strains.

Our metatranscriptomic results showed that *Desulfobulbaceae* microbes DB1 and DB2 were also activated during the EET-enhanced SP condition with a more positive surface potential, and strain DB1 performed EET to the solid electrode via MH-cytCs in addition to sulfur and acetate metabolisms (Fig. 5). These metabolisms have not been previously described for family *Desulfobulbaceae* isolates^41^,^50^,^51^. Dissimilatory sulfate and elemental sulfur reduction are well-known metabolic functions within family *Desulfobulbaceae* isolates; however, solid metal-oxide reduction as well as electrode respiration has only been reported in *Desulfobulbus propionicus* with lactate, propionate or pyruvate, but not acetate as an electron donor^50^. However, it should be noted that the strain DB1 is phylogenetic distinct from the previously reported *Desulfobulbaceae* isolates and does not associate to the known genera (Supplementary Fig. S14). The activities observed and described as a part of our stimulus-induced metatranscriptomic approach may help to address the reason why *Desulfobulbaceae* strains are more abundant than *Geobacter* strains in those sulfate-containing EET-active sediments.

The expanded use of this meta-omics approach could contribute toward revealing microbial functions and roles in geochemical cycling, eliciting effective strategies for applying microbial communities to industrial applications, and testing fundamental theories about microbial adaptation and evolution occurring in the environment^1^. While we have demonstrated the success of our approach for an enriched microbial community in a bioelectrochemical system, we propose that the same concepts and strategies can be applied to microbial communities in natural environments, although we recognize that there are different challenges (detailed discussion can be found in Supplementary Discussion).

Consequently, this new approach and resulting knowledge is a first step toward unveiling comprehensive microbial metabolic networks and addressing how microbial ecosystems function and maintain. The previously reported longer-term community dynamics trends^23^ correlate with the short-term stimulus-induced responses described here, which is a partial validation of these results. Further validation of our estimated metabolic network and metabolic switches will be conducted by meta-proteomic and metabolomic analyses of the community in response to the same EET stimulus. Additional stimulus-induced metatranscriptomics efforts associated with utilization of electron donors and estimated intermediates will also be explored. By combining these methods and associated results, we can expand our knowledge base and tools for describing complex microbial ecosystem dynamics in this era of rapid environmental change.

## Methods

### Metagenomic and metatranscriptomic samples

An electrogenic EET-active microbial community was established in an air-cathode microbial fuel cell (MFC) repeatedly fed with primary clarifier effluent from a municipal wastewater treatment plant^25^. The anode and cathode electrode were connected with an external resistor of 750 Ω during the 800 day MFC operation, and current generation was monitored as the function of microbial EET reaction. Two more MFCs fed with same wastewater were operated over 1 year in parallel for establishing comparable electrogenic microbial communities (MFC#2 and MFC#3). Community composition of these reactors was analyzed by using 16S rRNA amplicon sequencing as described elsewhere^52^.

The DNA and mRNA sequence raw reads yielded from our previous report^21^ were used in this study. In brief, three biofilm samples were harvested sequentially within a 3 hr time period after exposure to three different operating conditions. These include the standard MFC operation (MFC) with current density of 70 mA/m^2^, set-potential (SP) condition after controlling the electrode surface potential to +100 mV vs. standard hydrogen electrode with current density of 430 mA/m^2^, and open circuit (OC) condition with zero current production^21^. Both DNA and RNA of the anode-associated microbial community were coextracted using a MObio PowerBiofilm RNA Isolation Kit (MO BIO, San Diego, CA). The DNAs were sequenced using Illumina GAIIx (Illumina, San Diego, CA, USA) and 454 Titanium FLX (454 Life Sciences, CT, USA) platforms (Supplementary Table S1). The RNAs were sequenced using the GAIIx, and ribosomal RNAs were removed by *in silico* subtraction method^21^. The mRNA nucleotide sequences have been deposited in the NCBI Short Read Archive under accession number SRX189137-SRX189139.

### Revised assembly of metagenomic data sets

The revised *de novo* assembly of metagenomic sequences was conducted by CLC *de novo* Assembly Cell version 4.0 (CLCbio, Boston, MA, USA). All DNA data was mixed, assembled into contigs with scaffolding based on paired information using different kmer sizes (23, 33, 43, 53, and 63) and bubble lengths (100, 300, 500 and 700 bp). The assemblies were compared using total bases of the contigs, N50, and % mapped raw DNA reads to contigs (Supplementary Fig. S1). From a total of 20 sets, two assembly sets of bubble length 700 bp with kmer size 33 and 23 showed the best quality, and the assembly set with bubble length 700 bp and kmer size 33 was selected. The contigs over 500 bp in the selected assembly were used in subsequent analyses (Supplementary Table S1). The revised contigs have been deposited at DDBJ/EMBL/GenBank under the accession AMWB00000000. The version described in this paper is version AMWB02000000.

The contigs were functionally annotated as described previously^21^. Briefly, all contigs were taxonomically assigned based on most abundant taxonomic information of peptides in the contig^21^, and all open reading fames (ORFs) were functionally annotated based on KEGG orthologous (KO) groups^29^ using the KEGG Automatic Annotation Server^53^. ORFs were annotated to *c*-type cytochromes based on a CXXCH motif search, and 714 ORFs contained more than two occurrences of the motif indicating that these may be multi-heme *c*-type cytochromes (MH-cytCs). Conserved protein orthologous groups of *c*-type cytochromes^33^ were used for assigning to the *c*-type cytochrome family ID^21^.

### Bin-genome clustering

Bin-genomes were extracted by grouping contigs of the estimated dominant strains within the community. The specific values used for the Bin-genome clustering are summarized in Supplementary Table S2. The contig clusters were then refined by differential coverage method and tetra nucleotide frequency method^15^. To assess the genome “completeness” of Bin-genomes, we used 107 marker genes for *Bacteria* and 137 marker genes for *Archaea*^21^. Percentages of single-copied housekeeping genes were used to estimate the genome completeness and mixture of multiple genomes for each Bin-genome. A principal component analysis (PCA) diagram of the marker gene existence matrix of *Bacteria* was constructed using XLSTAT (Addinsoft, New York, NY, USA) to identify representative marker genes. Quality assessment for the Bin-genomes was conducted by QUAST tool^54^ and finishing standards by provisional HMP Consortium definitions^26^. Ribosomal RNA-associated regions were assigned to phylotypes (clone IDs) by using the BLAST program to link Bin-genomes to phylotypes from the 16S rRNA clone analyses^21^. If direct linkages were not established, associations were estimated by comparing phylogenetic positions of 16S rRNA and three housekeeping proteins (RplE, NusA, and PheS) using phylogenetic trees created by the neighbor-joining algorithms in CLC Genomics Workbench version 5.0.

### Metagenomic community composition analysis

Relative frequencies of Bin-genomes were determined using several different methods. Sixteen single-copied housekeeping genes were selected from the PCA diagram, and the relative frequencies of each Bin-genome were determined from the mean coverage of the contigs that include ORFs of each selected housekeeping gene. The average of the relative frequencies for the marker genes was calculated as community composition. The community composition was also analyzed based on metagenomic raw read frequencies.

### Read mapping of raw reads to ORFs

RPKM values, Reads Per Kilobase per Million mapped reads^55^, for both DNA and mRNA samples under three different operational conditions (MFC, SP, and OC) were generated by the RNA-Seq Analysis pipeline in CLC Genomics Workbench (version 6.5), and used to analyze ORF frequency (DNA-RPKM) and gene expression levels (mRNA-RPKM). All 359,891 ORFs were used as references, and read mapping was conducted using 0.5 as the minimum length and 0.95 as the minimum similarity fractions. The calculated mean of the DNA-RPKM for three conditions was used to determine gene existence level of each ORF, while mRNA-RPKM values for each condition were normalized by using the ratio between corresponding DNA-RPKM for each condition and the mean of DNA-RPKM.

### Selection of marker genes for analyzing cell activity and metabolisms

Microbial cell activity- and metabolism-associated marker genes were selected from the KEGG module or KEGG pathway databases^29^. Selected marker gene families for cell activity include central dogma-associated KEGG modules that are essential for life, ATP synthase that is essential for energy production, and stress response associated genes such as superoxide dismutase. Selected marker gene families for microbial metabolisms include KEGG modules associated with glycolysis, TCA cycle, a wide selection of genes related to different forms of prokaryotic respiration, and manually selected genes associated with production of fermentation byproducts and potential substrate transporters, all of which are of potential importance for anaerobic microbial life. Detailed criteria for selection are described in Supplementary Methods. The KOs used for analyses are summarized in Supplementary Data 1 for cell activity and Supplementary Data 2 for microbial metabolisms, along with mRNA-RPKM for each condition and mean DNA-RPKM. The gene expression levels were normalized by dividing the mRNA-RPKM by the DNA-RPKM (mRNA/DNA ratio). From the full list of marker gene families for microbial metabolisms, key KEGG module and KEGG orthology (Fig. 4) were selected for describing metabolic pathway networks as described above.

## Additional Information

**How to cite this article**: Ishii, S. *et al*. Microbial metabolic networks in a complex electrogenic biofilm recovered from a stimulus-induced metatranscriptomics approach. *Sci. Rep*. **5**, 14840; doi: 10.1038/srep14840 (2015).

## Supplementary Material

Supplementary Information

Supplementary Data 1

Supplementary Data 2

Supplementary Data 3

Supplementary Data 4

## Figures and Tables

**Table 1:** Summary of Bin-genomes clustered from metagenomic assembly.

Bin-genome ID	Taxonomy	Core gene-basis frequency (%)^a^	Genome size (Mbp)	No. of gene/ ORF^b^	% Complete- ness^c^		No. of contigs	GC content (%)
MS1	*Methanosarcinaceae*	**22.7** ± 2.5	2.21	2155	**90**	**[HQD]**	171	44
DB1	*Desulfobulbaceae*	**7.2** ± 0.7	3.51	3188	**95**	**[HQD]**	**79**	54
Bet1	*Betaproteobacteria*	**3.3** ± 0.5	2.59	3020	87	[SD]	487	64
Bac1	*Bacteroidetes*	**3.1** ± 0.3	2.98	2514	**96**	**[HQD]**	**86**	50
DF1	*Desulfobacteraceae*	**2.7** ± 0.5	3.52	3272	**90**	**[HQD]**	166	57
Unc1	Unassigned	**2.7** ± 1.1	3.60	3081	80	[SD]	81	59
DM1	*Desulfuromonadaceae*	**2.4** ± 0.6	2.86	3361	84	[SD]	908	60
Unc2	Unassigned	**2.2** ± 0.9	2.53	2634	82	[SD]	**73**	36
DB2	*Desulfobulbaceae*	**1.8** ± 0.3	2.49	2217	**93**	**[HQD]**	114	50
Bac2	*Bacteroidetes*	**1.7** ± 0.2	4.59	3748	**96**	**[HQD]**	218	44
DF2	*Desulfobacteraceae*	**1.5** ± 0.5	3.35	2967	78	[SD]	262	48
Chl1mix^d^	*Chloroflexi* etc.	**3.9** ± 1.1	3.88	3715	190^d^	[mix]	302	54
DM2mix^d^	*Geobacteaceae* etc.	**2.9** ± 0.7	7.73	10512	347^d^	[mix]	3843	58

^a^Averaged frequency within the metagenome was calculated based on the coverage of 16 universal single-copied core genes (Mean ± SD).

^b^Numbers of ORFs have potential errors in metagenomic ORF calling because of incomplete assemblies.

^c^Values were calculated from frequency of KO assignment to universal single-copied gene family lists (Supplementary Table S3 and S4). Quality of draft genomes (HQD, High-quality draft; SD, Standard draft; mix, mixture of two or more genomes) were assessed by HMP criteria (Supplementary Table S6)^26^.

^d^Chl1mix was considered as mixture of two genomes, while DM2mix was considered as mixture of over three genomes. Those bin-genomes were not used for subsequent metabolic network analyses.

**Table 2:** Summary of parameters for stimulus-induced metatranscriptomic analyses.

Condition^a^	Time after stimulus^b^	Current density (mA/m^2^)^c^	Total mRNA reads^d^	Mapped mRNA reads^e^	
				All ORFs^f^	Bin-genome ORFs^g^
MFC	5 hr	70	467,556	196,392 (42%)	125,024 (27%)
SP	45 min	430	521,036	222,585 (43%)	147,875 (28%)
OC	45 min	0	513,056	231,056 (45%)	153,834 (30%)

^a^MFC, microbial fuel cell operation with 750 Ω external resistor; SP, set potential operation to control anode surface potential of +100 mV vs SHE; OC, open circuit operation to disconnect electrical circuit.

^b^Total RNAs were extracted under steady-state MFC condition, and stimulus-induced SP/OC conditions.

^c^Anodic current density normalized by projected anode surface area.

^d^mRNA was obtained by rRNA subtraction from total raw RNA reads using SILVA database.

^e^mRNA was mapped to ORFs called from contigs with the parameter as 0.95 of identity and 0.7 of length coverage.

^f^Numbers of mRNA reads mapped to all ORFs. Parenthesis indicates % mapped to all ORFs.

^g^Numbers of mRNA reads mapped to ORFs from 13 bin-genomes. Parenthesis indicates % mapped to the ORFs.
