# Supplementary material for: Microbial metabolic networks in a complex electrogenic biofilm recovered from a stimulus-induced metatranscriptomics approach
Source: Sci Rep. 2015 Oct 7;5:14840. doi: 10.1038/srep14840 (PMC4595844; doi:10.1038/srep14840)
Supplement: Supplementary Information [file srep14840-s1.pdf]

Microbial metabolic networks in a complex electrogenic biofilm recovered from a stimulus-induced metatranscriptomics approach

Shun'ichi Ishii*, Shino Suzuki*, Aaron Tenney, Trina M. Norden-Krichmar,
Kenneth H. Nealson, Orianna Bretschger

* Corresponding authors
SI, E-mail: sishii@jamstec.go.jp; Tel: +81-88-878-2279; Fax: +81-88-878-2192
SS, E-mail: sisuzuki@jamstec.go.jp; Tel: +81-88-878-2224; Fax: +81-88-878-2192

SUPPLEMENTARY INFORMATION

Table of Contents

SUPPLEMENTARY FIGURES **2**

SUPPLEMENTARY TABLES **16**

SUPPLEMENTARY DISCUSSION **31**

Characterization of thirteen bin-genomes 31
Accurate community composition for complex microbial community 31
Stimulus-induced metatranscriptomics for natural environmental community 32

SUPPLEMENTARY METHODS **33**

Selection of marker genes for analyzing cell activity and microbial metabolisms 33

SUPPLEMENTARY REFERENCES **34**

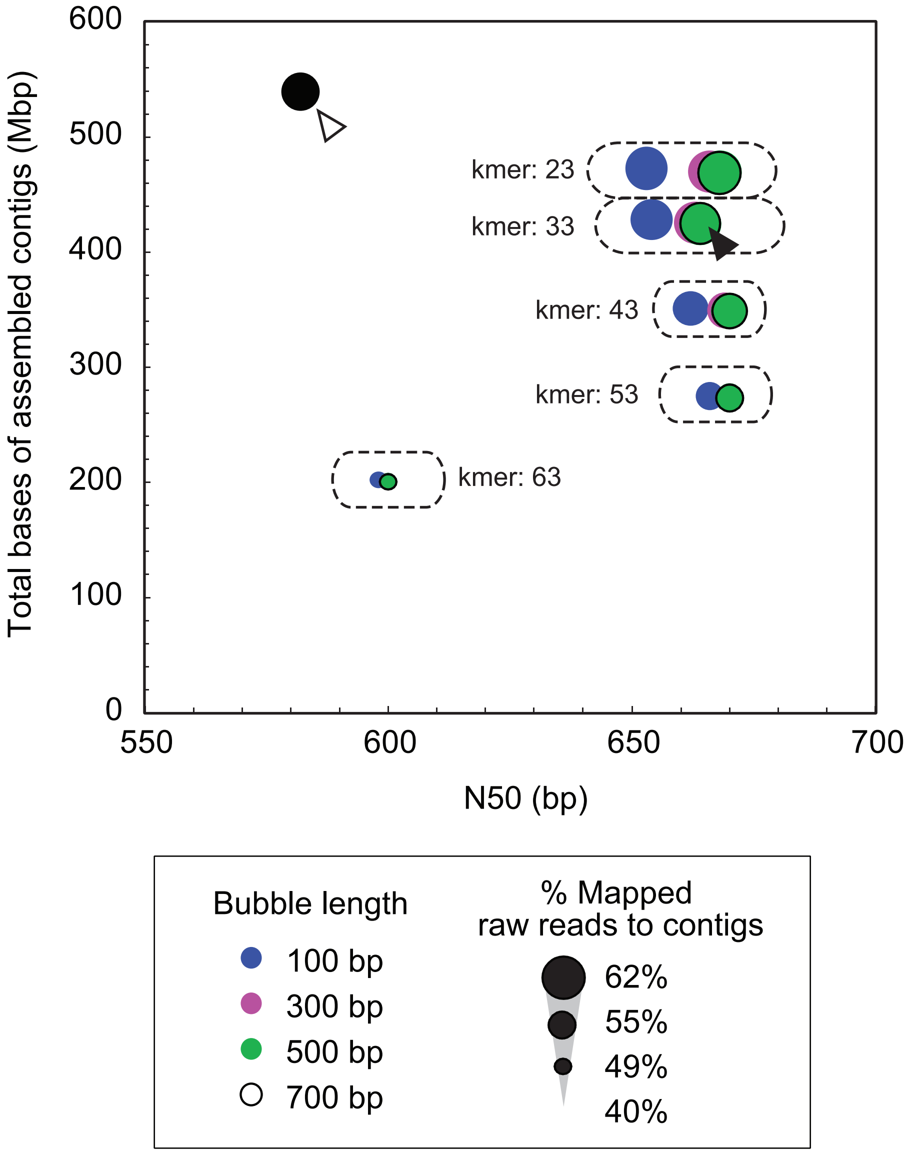

Supplementary Fig. S1 | Metagenomic assembly metrics with the various parameters on *de novo* assembly software.

Three metagenomic assembly metrics, total bases (y-axis), N50 (x-axis), and % mapped raw reads back to the assmbled contigs (size of bubble), are shown with different parameters of both kmer size (dashed line) and bubble length (color) in CLC *de novo* assembly cell 4.0. Open arrowhead indicates an assembly used in previous study (Ishii et al. 2013. Nature Commun. 4: 1601). Filled arrowhead indicates an assembly used in this study.

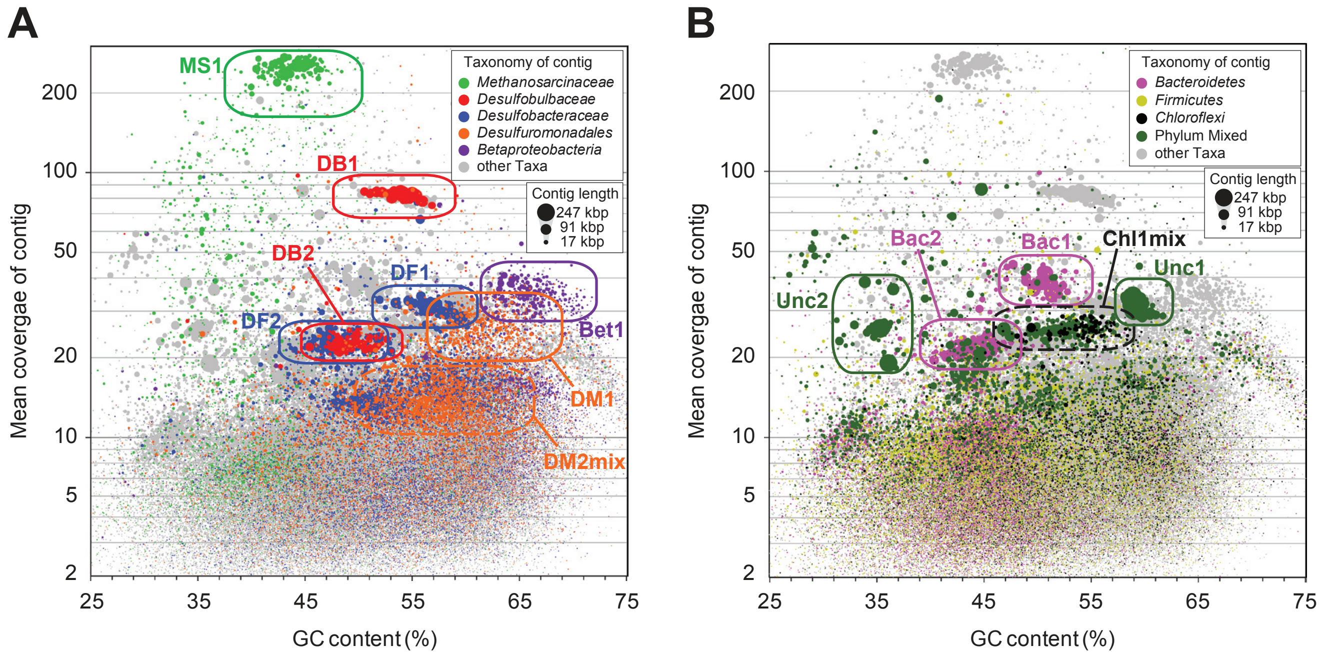

Supplementary Fig. S2 | Identified Bin-genomes of dominant strains within the EET-active microbial community.

Panel A shows Bin-genome clusters (colored rounded rectangles) for taxa related to Phylum Proteobacteria and Family Methanosacrinaceae, while panel B shows Bin-genome clusters for the other taxa. Bin-genomes (ID described near rounded rectangles) were established using the estimated taxonomic classification of contigs (color of dots), contig lengths (size of dots), GC content of contigs (%), and mean coverage of contig.

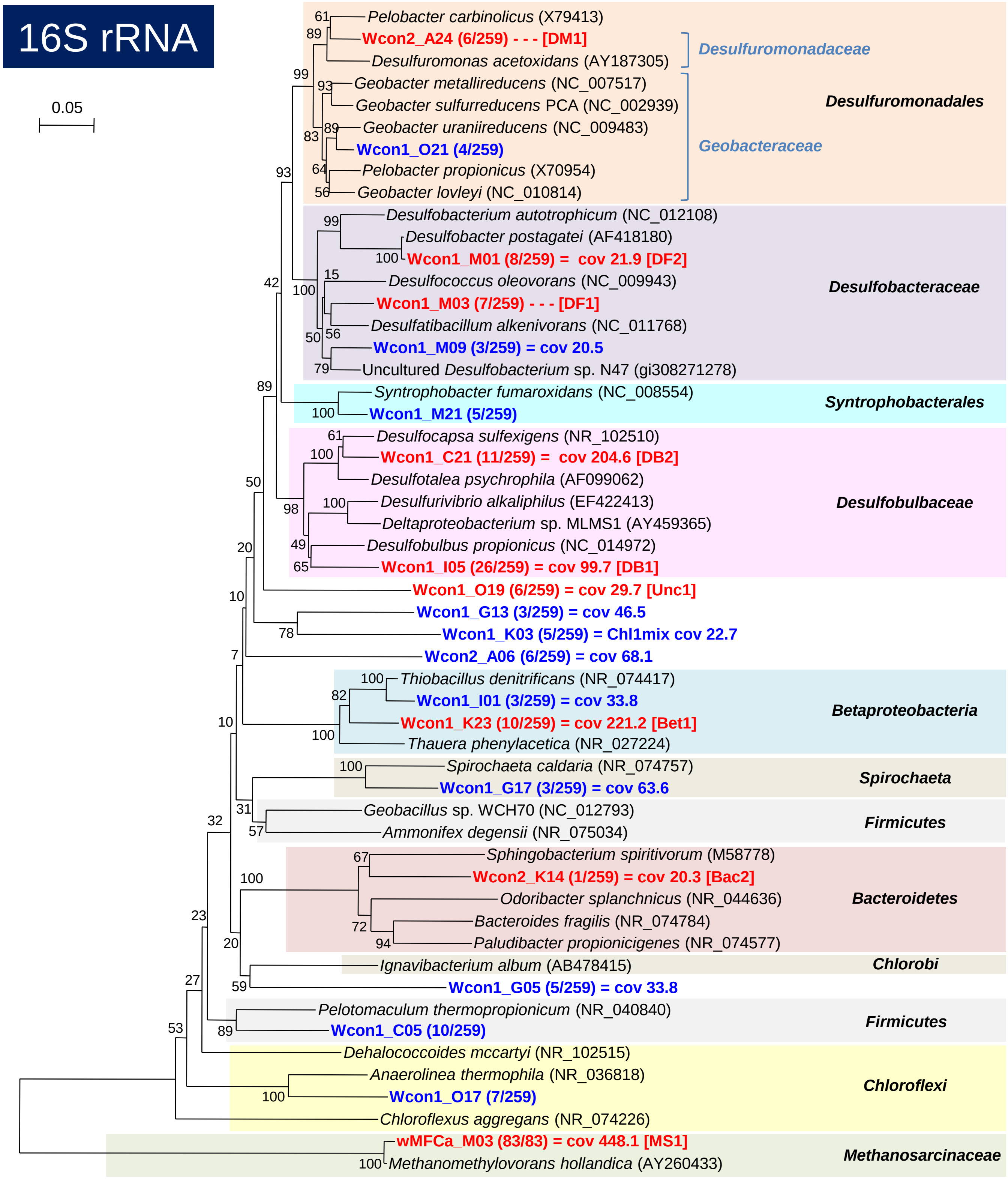

Supplementary Fig. S3 | Neighbor-joining phylogenetic tree of 16S rRNA showing positions of abundant phylotypes (clones >3) from 16S rRNA clone analysis.

The 21 abundantly observed 16S rRNA genes in the community are described as "clone ID (numbers in the clone library / total clones analyzed) = contig coverage value if the 16S rRNA sequence was observed in the metagenomic assembly [Bin-genome ID]". Phylogenetic position of the Bin-genome DB1 and DF1 were estimated by comparison between topologies of three housekeeping genes as described in Supplementary Figs. S3-S5. Bootstrap values (100 trials) are described at each branch point. Accession numbers of reference sequences are indicated in parentheses.

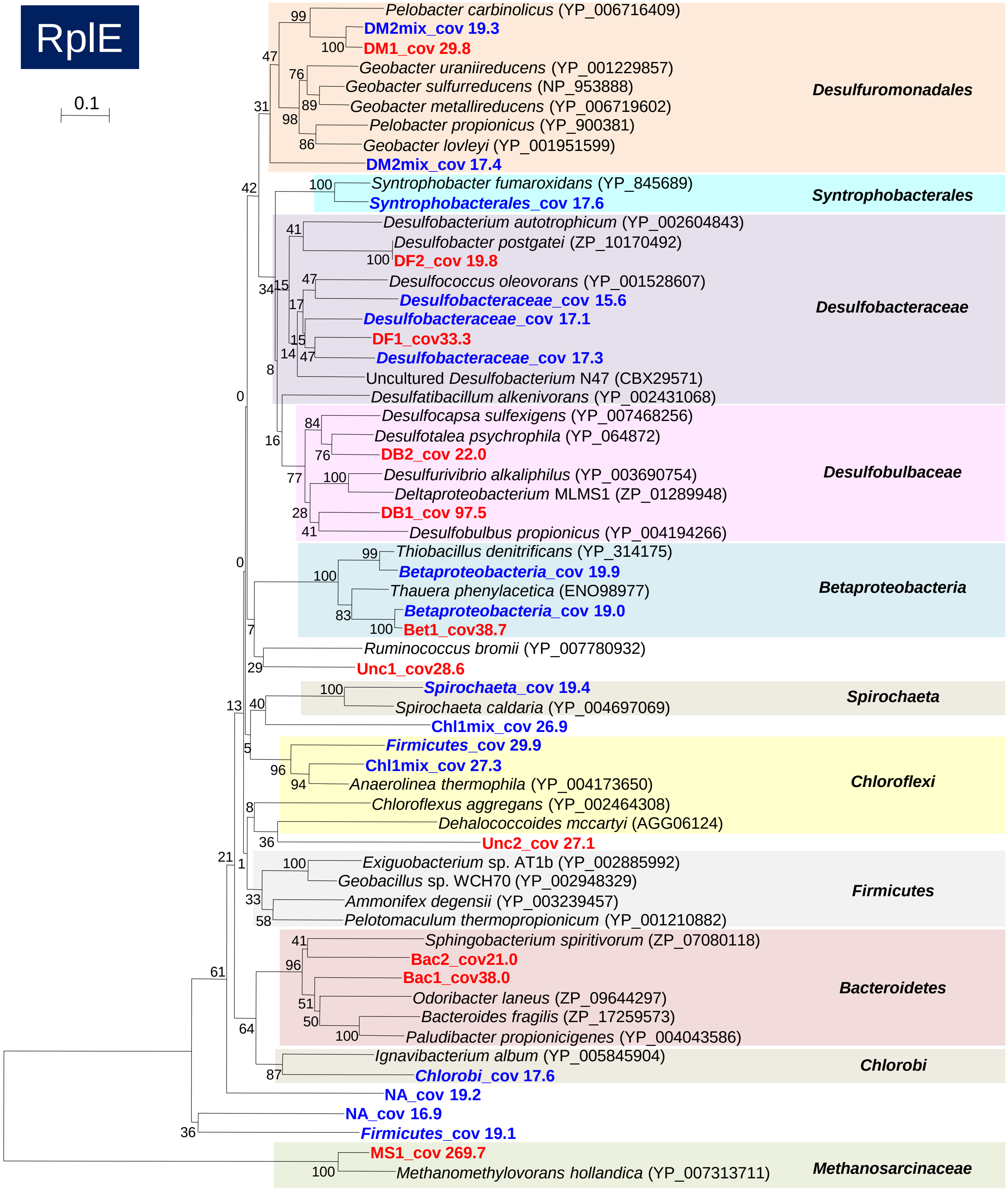

Supplementary Fig. S4 | Neighbor-joining phylogenetic tree of RplE peptides showing positions of abundant phylotypes (contig coverage >15) including bin-genomes.

The 26 abundantly observed RplE peptides in the community are described as "bin-genome ID (or taxonomic assignment of the contig)_contig coverage value". Bootstrap values (100 trials) are described at each branch point. Accession numbers of reference sequences are indicated in parentheses.

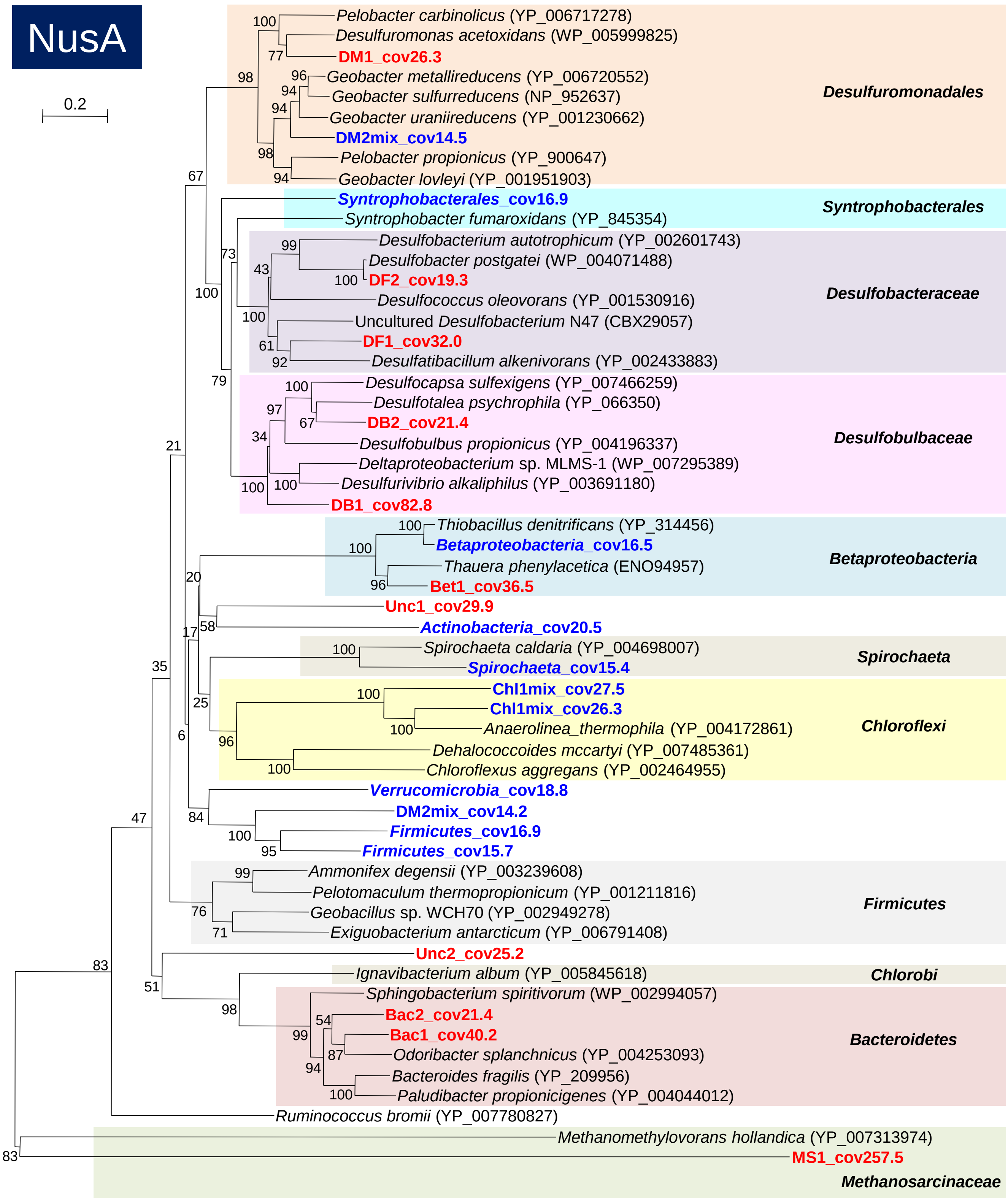

Supplementary Fig. S5 | Neighbor-joining phylogenetic tree of NusA peptides showing positions of abundant phylotypes (contig coverage >13) including bin-genomes.

The 22 abundantly observed NusA peptides in the community are described as “bin-genome ID (or taxonomic assignment of the contig)_contig coverage value”. Bootstrap values (100 trials) are described at each branch point. Accession numbers of reference sequences are indicated in parentheses.

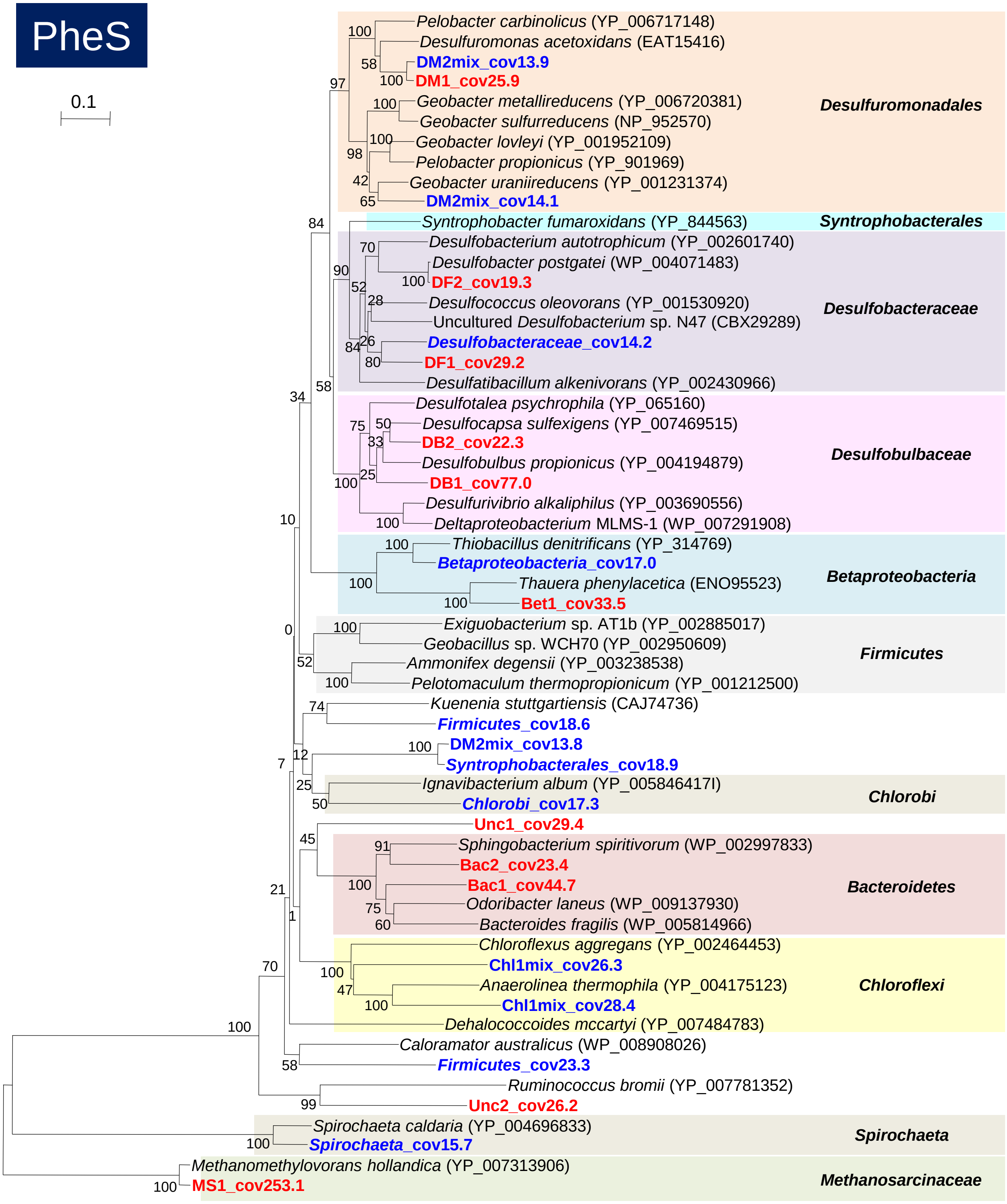

Supplementary Fig. S6 | Neighbor-joining phylogenetic tree of PheS peptides showing positions of abundant phylotypes (contig coverage >13) including bin-genomes.

The 23 abundantly observed PheS peptides in the community are described as “bin-genome ID (or taxonomic assignment of the contig)_contig coverage value”. Bootstrap values (100 trials) are described at each branch point. Accession numbers of reference sequences are indicated in parentheses.

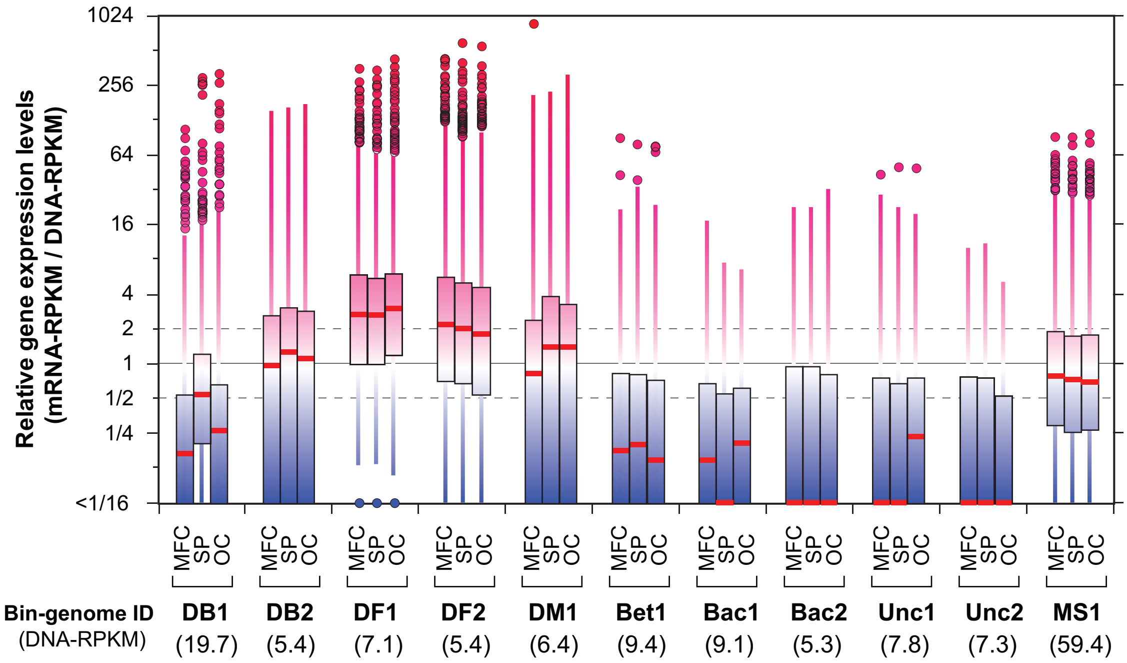

Supplementary Fig. S7 | Gene expression levels of each bin-genome within the EET-active microbial community.

Box-plot diagram showing the normalized gene expression levels of CDSs in each bin-genome for three different operational conditions (MFC, SP, and OC) based on DNA-RPKM (parenthesis below bin-genome ID). The box signifies the upper and lower quartiles, the bar signifies the upper and lower adjacent values, and the median is represented by a red line within the box for each sample. CDSs showed over upper adjacent value and under lower adjacent value are depicted by circles.

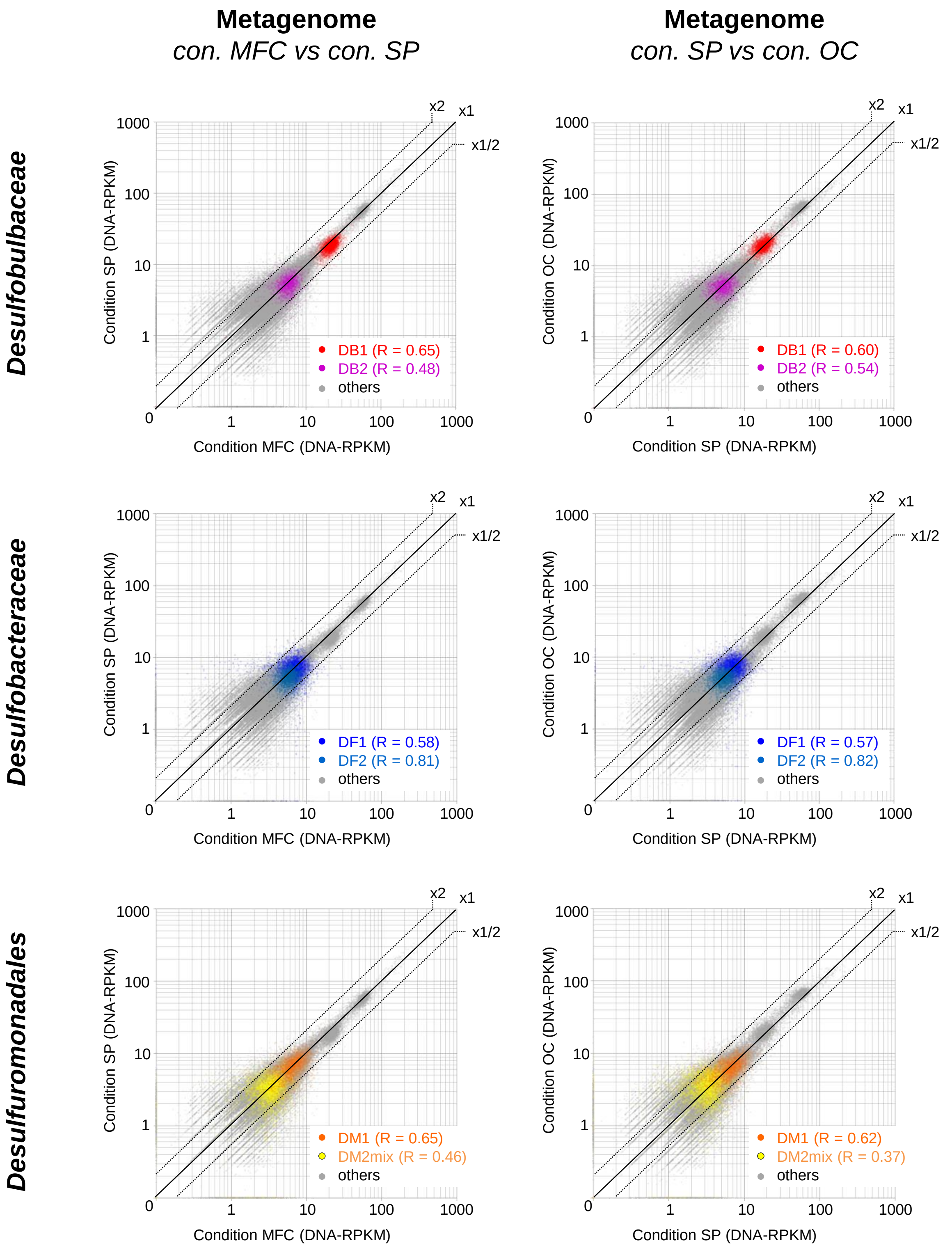

Supplementary Fig. S8. Scatter plots of CDSs for *Deltaproteobacterial* bin-genomes with DNA frequency between two operational conditions.

Left panels show the relationship between MFC and SP conditions. Right panels show the relationship between SP and OC conditions. The correlation coefficient (R) is shown.

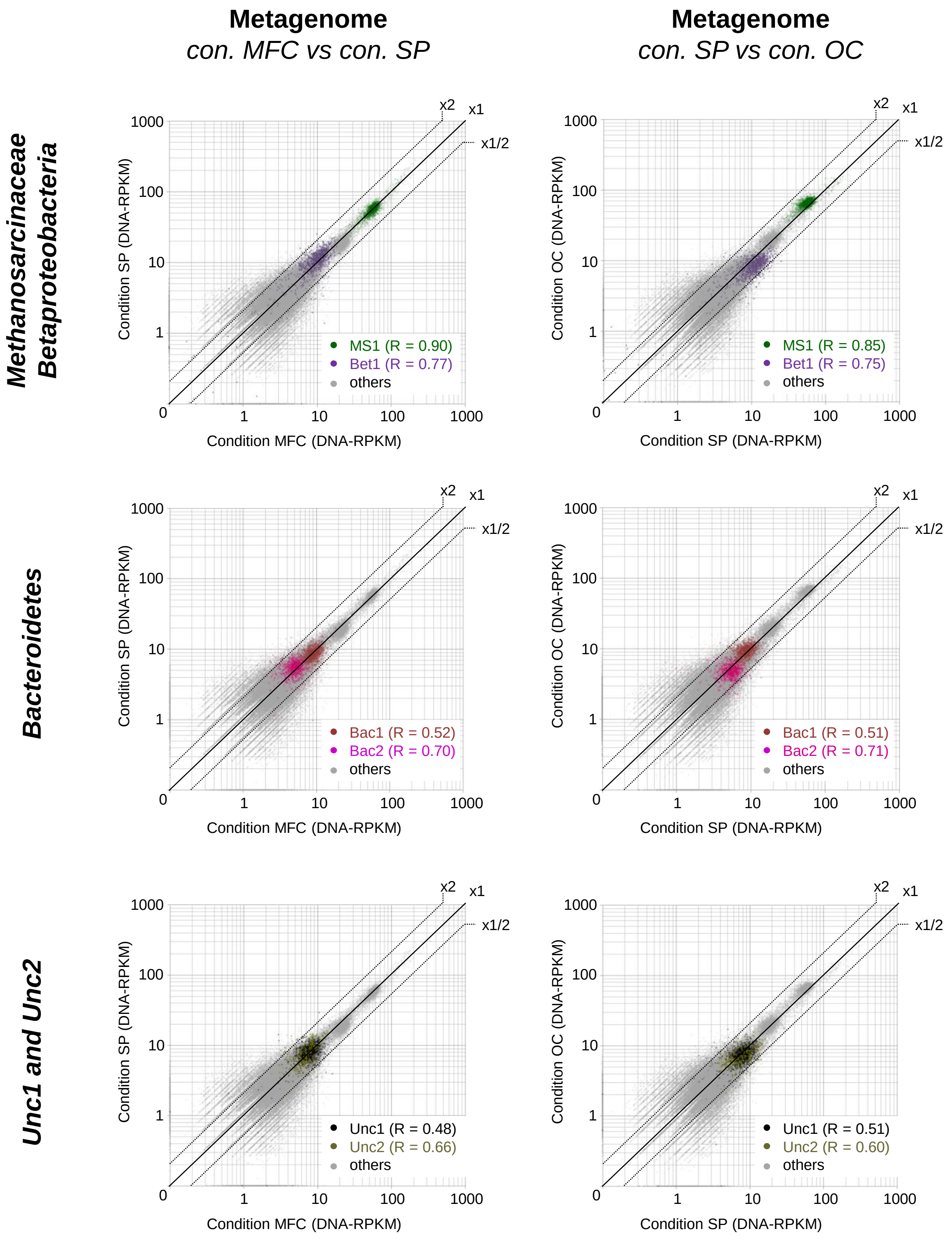

Supplementary Fig. S9. Scatter plots of CDSs for non-*Deltaproteobacterial* bin-genomes with DNA frequency between two operational conditions.

Left panels show the relationship between MFC and SP conditions. Right panels show the relationship between SP and OC conditions. The correlation coefficient (R) is shown.

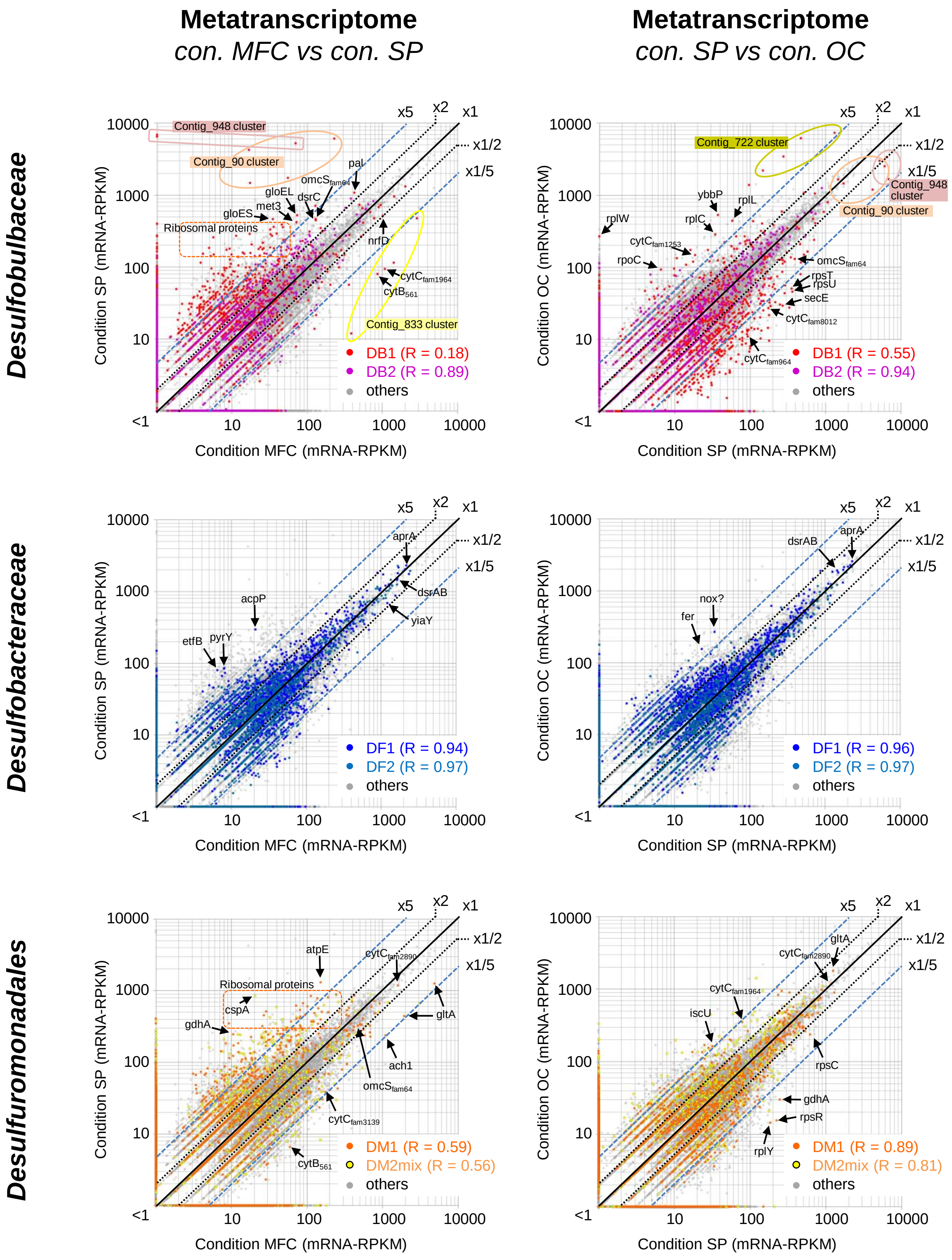

Supplementary Fig. S10. Scatter plots of CDSs for *Deltaproteobacterial* bin-genomes with mRNA frequency between two operational conditions.

Left panels show the relationship between MFC and SP conditions. Right panels show the relationship between SP and OC conditions. The correlation coefficient (R) is shown.

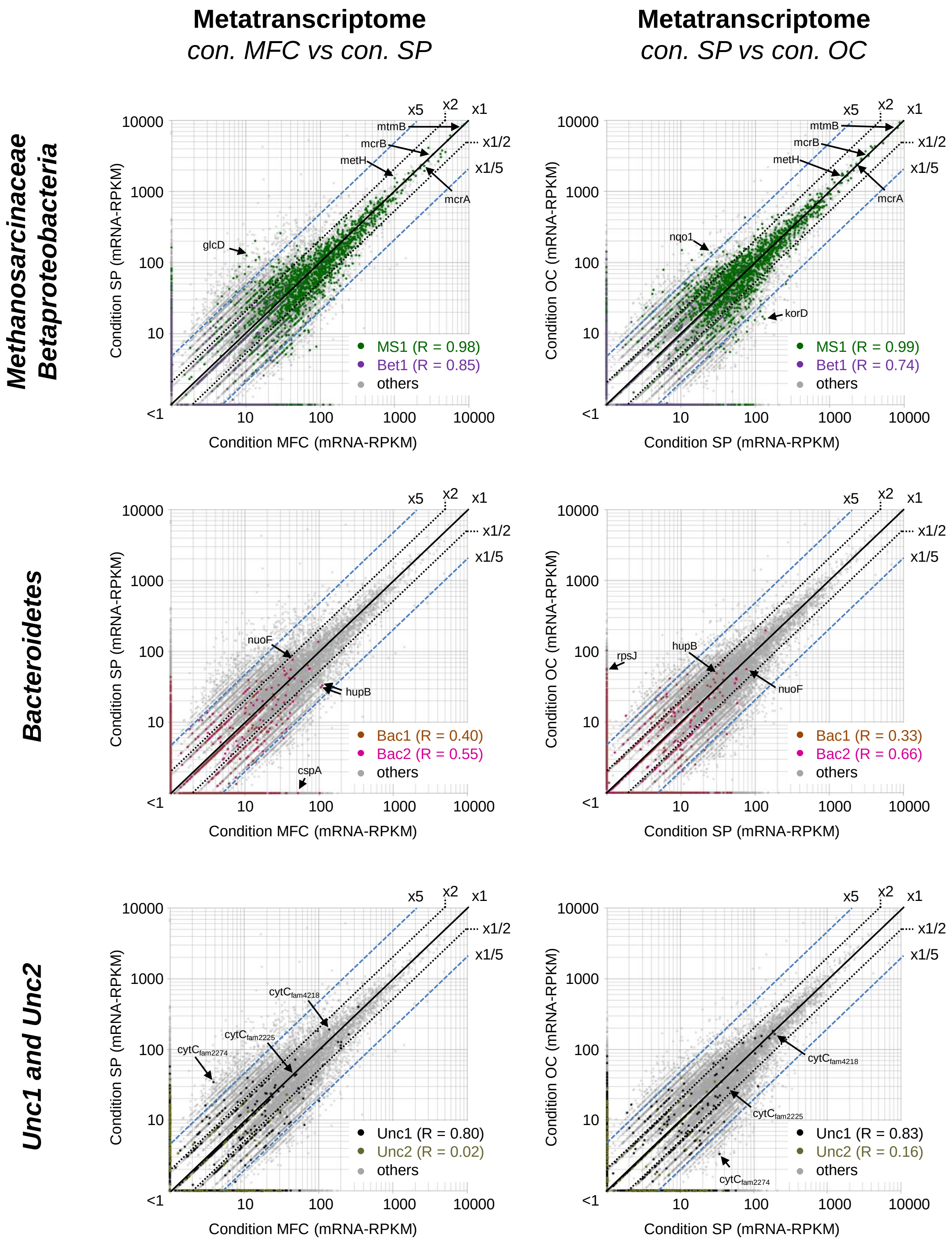

Supplementary Fig. S11. Scatter plots of CDSs for non-*Deltaproteobacterial* bin-genomes with mRNA frequency between two operational conditions.

Left panels show the relationship between MFC and SP conditions. Right panels show the relationship between SP and OC conditions. The correlation coefficient (R) is shown.

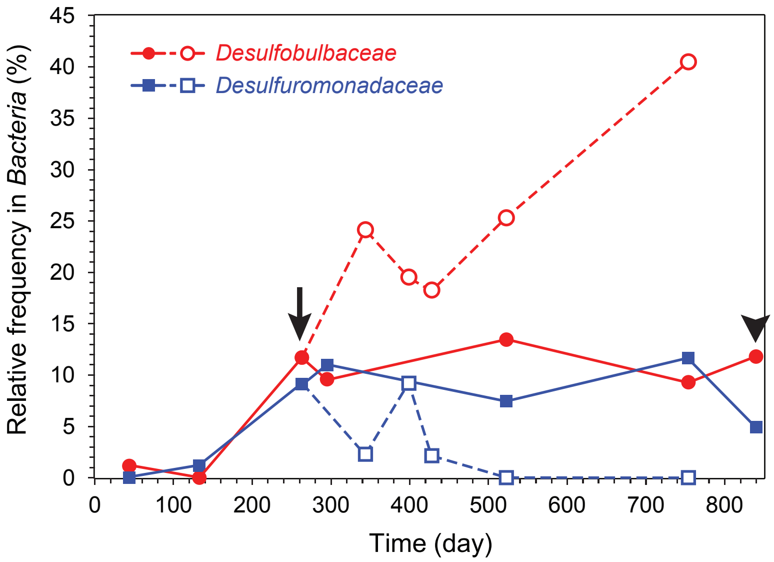

Supplementary Fig. S12 | Comparison of long-term community dynamics between *Desulfobulbaceae* and *Desulfuromonadaceae* after SP stimulus.

Longer-term population dynamics of those two EET-responsive families, family *Desulfobulbaceae* (red circle) and family *Desulfuromonadaceae* (blue square) suggested from the stimulus-induced metatranscriptomics was analyzed by bacterial 16S rRNA-basis community analyses of the anode-associated electrogenic biofilm, which was reported from the previous studies (Ishii et al. 2013 Water Res. 47:7120-30). The MFC was repeatedly fed with wastewater over 800 days (solid symbols), and the stimulus-induced metatranscriptomic analysis of this study was conducted at day 840 of the operation (arrowhead). The intermittent SP operation was started from day 264 by sub-passage to new reactor (arrow), and continuous SP operation was started from day 500 (open symbols).

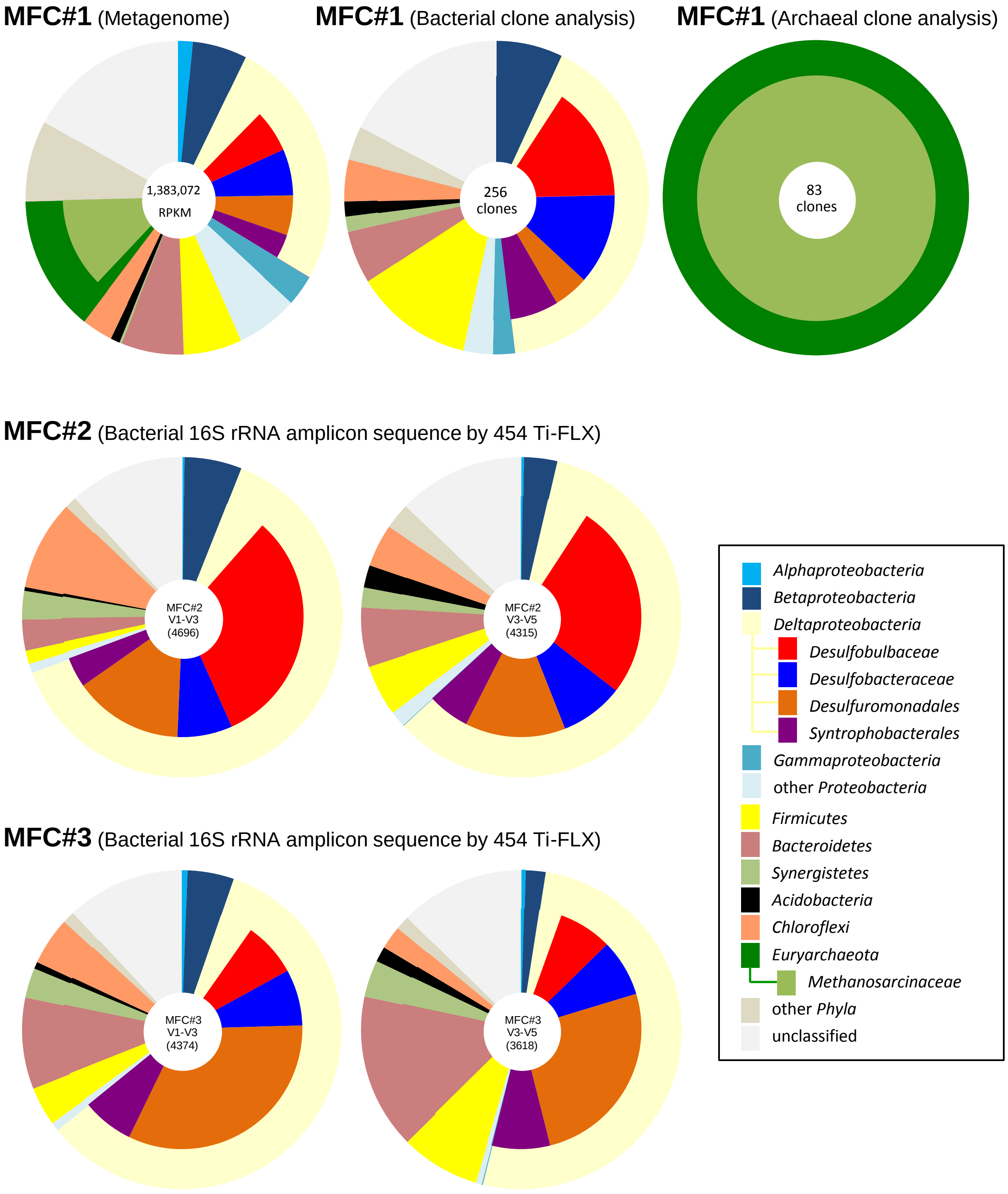

Supplementary Fig. S13 | Electrogenic microbial community compositions in three different wastewater-fed microbial fuel cells.

MFC#1 was analyzed based on the metagenomic DNA frequency (DNA-RPKM), 16S rRNA clone analyses (total number of sequenced clone is described in center circles) for both *Bacteria* and *Archaea* which are described in previous study (Ishii et. al. 2013. Nat Commun 4:1601). MFC#2 and MFC#3 were analyzed based on 454 pyrosequencing of V1-V3 region or V3-V5 region of 16S rRNA amplicon (number of sequenced reads are described in paraphrase of the center circle), which method is described in previous work (The Human Microbes Project Consortium, 2012. Nature 486: 215–221).

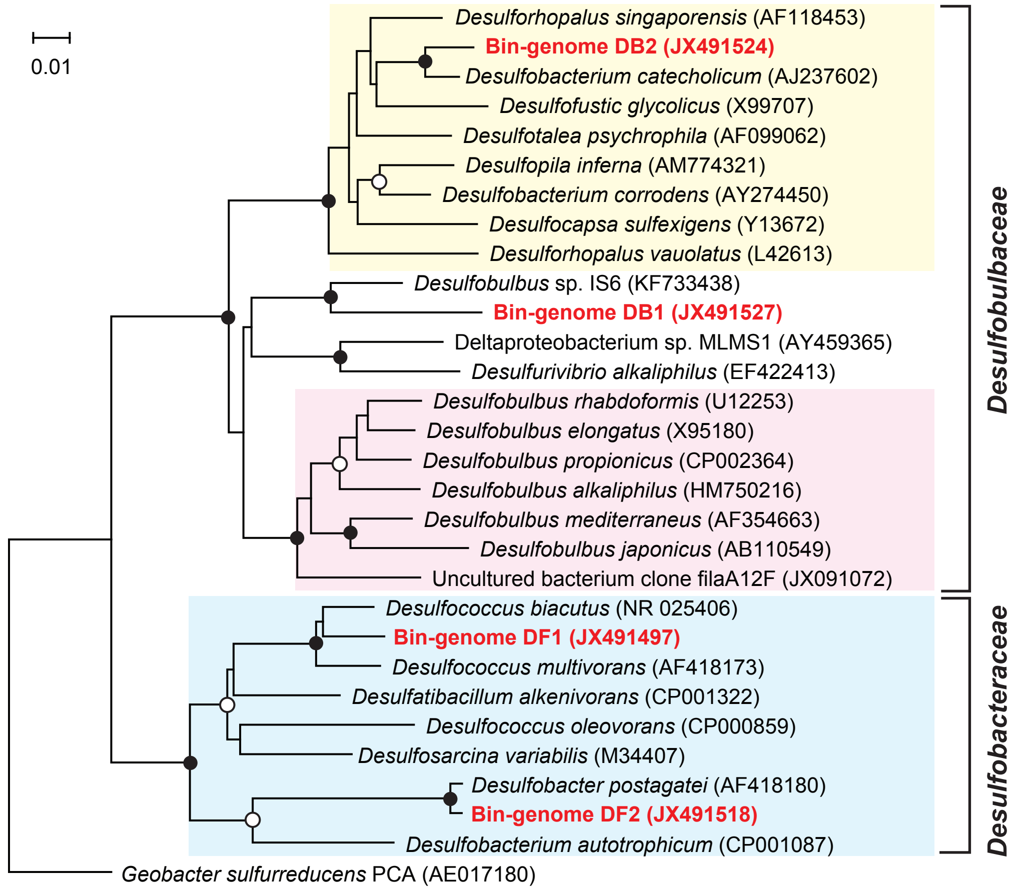

Supplementary Figure S14. A neighbor-joining tree showing phylogenetic positions of major *Desulfobacterales* bin-genomes representing anodic microbial populations.

Branch points supported with bootstrap values (100 trials) of >90% are indicated with closed circles, while those between 70% and 90% are indicated with open circles. Accession numbers of reference sequences are indicated in parentheses.

Supplementary Table S1. Statistical parameters of metagenomic assemblies .

Step ID	Length cutoff	No. of reads[a]	Reads Mapped	% Mapped	No. Contigs	Total Bases (bp)	Max contig size (bp)	N50 (bp)	No. contigs >1k
Raw sequence reads									
454 Ti (mated pair)	-	1,152,579	-	-	-	184,768,596	-	-	-
Illumina (fragment)	-	38,353,992	-	-	-	3,835,399,200	-	-	-
Illumina (paired end)	-	69,613,842	-	-	-	6,961,384,200	-	-	-
Multi-step assembly[b]	> 200 bp	38,353,992	22,448,289	58.5%	1,005,833	539,435,694	233,369	582	73,068
	> 500 bp	38,353,992	18,479,506	48.2%	224,995	298,152,130	233,369	1643	73,068
CLC assembling cell 4.0[c]	> 200 bp	96,354,452	60,027,569	62.3%	717,513	424,568,988	280,490	664	57,714
	**> 500 bp**	**96,354,452**	**49,837,201**	**51.7%**	**169,740**	**248,451,270**	**280,490**	**2021**	**57,714**

[a] Numbers of reads to perform mapping raw reads back to the assembled contigs (with length cutoff 0.8 and similarity cutoff 0.8).

[b] Multi-step assembly procedure is described in the previous paper (Ishii et al. Nature Commun. 4: 1601).

[c] CLC *de novo* assembler using all three phyrosequencing data with word length of 33 and bubble length of 800 (This study).

Supplementary Table S2. summary of criteria for contig groupings used to establish bin-genomes

Strain	Predicted taxonomy	Relative abundance (Mean coverage)[a]	GC content (%)	Contig length cutoff (bp)
**Bin-genome MS1**	Phylum *Euryarchaeota*	196 - 500	-[c]	-
**Bin-genome Bac1**	Phylum *Bacteroidetes*	30 - 50	45 - 55	-
**Bin-genome Bac2**	Phylum *Bacteroidetes*	18 - 26	37 - 50	-
**Bin-genome Bet1**	Class *Betaproteobacteria*	23 - 50	>59	-
**Chl1mix**	Phylum *Mixed*[b]<br>Phylum *Chloroflexi*	20 - 32<br>22 - 28	40 - 60<br>45 - 57	-
**Bin-genome Unc1**	Phylum *Mixed*[b]	28 - 40	>56	-
**Bin-genome Unc2**	Phylum *Mixed*[b]	18 - 40	<40	-
**Bin-genome DM1**	Order *Desulfuromonadales*	20 - 50	-	-
**DM2mix**	Order *Desulfuromonadales*	10 - 20	-	-
**Bin-genome DB1**	Order *Desulfobacterales*<br>Class *Deltaproteobacteria*	70 - 90	-	-<br>>5000
**Bin-genome DB2**	Family *Desulfobulbaceae*<br>Family *Mixed* in *Desulfobacterales*	20 - 30	42 - 53<br>>50	-
**Bin-genome DF1**	Order *Desulfobacterales*<br>and not Family *Desulfobulbaceae*	26 - 35	<45	-
**Bin-genome DF2**	Family *Desulfobacteraceae*<br>Family *Mixed* in *Desulfobacterales*	19 - 30<br>20 - 30	42 - 53<br><50	-

[a] Relative frequency within the metagenome was calculated by read mapping of raw reads to contigs.
[b] Contigs assigned to phylum "*Mixed*", and also not assigned to kingdom *Virus*.
[c] Not applied for the contig grouping.

Supplementary Table S3. Single-copied housekeeping gene list for validation of bin-genome association.

Number of ORF annotated to specific KO within bin-genomes[g]: 0 | 1 | 2 | 3 | >4

KO	Gene symbol	Description	Average peptide length ± SD	House keeping (%)[a]	Single copied (%)[b]	Bin-genome: Bac1	Bac2	Bet1	Unc1	Unc2	DM1	DB1	DF1	DB2	DF2	MS1[i]	Chl1mix	DM2mix
K02982	rpsC[cd]	ribosomal protein S3 [S10 operon]	237 ± 23	98.8	99.8	1	1	1	1	1	1	1	1	1	1	1	2	3
K02988	rpsE[cd]	ribosomal protein S5 [rpc operon]	180 ± 23	98.7	99.9	1	1	1	1	1	1	1	1	1	1	1	1	2
K02994	rpsH[c]	ribosomal protein S8 [rpc operon]	132 ± 17	98.1	99.9	1	1	1	1	1	1	1	1	1	1	1	2	3
K02948	rpsK[cd]	ribosomal protein S11 [alpha operon]	130 ± 7	98.0	99.8	1	1	1	1	1	1	1	1	1	1	1	1	2
K02952	rpsM[cd]	ribosomal protein S13 [alpha operon]	124 ± 11	98.5	99.8	1	1	1	1	1	1	1	1	1	1	1	1	2
K02931	**rplE[cde]**	ribosomal protein L5 [rpc operon]	182 ± 8	98.3	99.6	1	1	1	1	1	1	1	1	1	1	1	2	5
K02933	rplF[cd]	ribosomal protein L6 [rpc operon]	178 ± 7	98.7	99.8	1	1	1	1	1	1	1	1	1	1	1	2	2
K02874	rplN[cd]	ribosomal protein L14 [rpc operon]	123 ± 5	98.3	99.6	1	1	1	1	1	1	1	1	1	1	1	2	3
K03687	grpE[c]	co-chaperone GrpE	203 ± 31	97.6	97.3	1	1	1	1	1	1	1	1	1	1	1	2	3
K02600	**nusA[cde]**	transcription termination factor NusA	424 ± 100	97.6	100.0	1	1	1	1	1	1	1	1	1	1	1	2	4
K03979	cgtA[c]	Obg family GTPase CgtA	398 ± 56	97.6	99.8	1	1	1	2	0	1	1	1	1	1	1	2	5
K03106	ffh[c]	signal recognition particle subunit SRP54	469 ± 34	96.7	99.9	1	1	1	2	1	1	1	1	2[h]	0	1	1	5
K01874	fmt[c]	methionyl-tRNA formyltransferase	624 ± 75	98.0	94.9	1	1	1	1	1	1	1	1	1	1	1	2	4
K01875	serS[c]	seryl-tRNA synthetase	429 ± 11	99.6	97.7	1	1	1	1	1	1	1	1	1	1	1	2	4
K01889	**pheS[ce]**	phenylalanyl-tRNA synthetase, alpha subunit	355 ± 47	97.9	99.2	1	1	1	1	2	1	1	1	1	1	1	2	4
K01873	valS[c]	valyl-tRNA synthetase	904 ± 79	99.7	98.2	1	1	1	1	1	2[h]	1	1	1	1	1	2	7
K02863	rplA[d]	ribosomal protein L1	231 ± 9	97.8	99.9	1	1	1	0	1	1	1	1	1	1	1	2	3
K02886	rplB[d]	ribosomal protein L2 [S10 operon]	274 ± 11	98.8	99.7	1	1	1	1	1	1	1	1	1	1	1	2	2
K02906	rplC[d]	ribosomal protein L3 [S10 operon]	215 ± 16	98.1	99.7	1	1	1	1	1	1	1	1	1	1	1	1	2
K02864	rplJ	ribosomal protein L10	172 ± 10	99.1	99.9	1	1	0	0	1	1	1	1	1	1	1	2	2
K02867	rplK[d]	ribosomal protein L11	144 ± 24	98.2	99.5	1	1	1	0	1	2[h]	1	1	1	1	1	2	4
K02871	rplM[d]	ribosomal protein L13	147 ± 20	98.2	99.9	1	1	1	1	1	1	1	1	1	1	1	2	4
K02876	rplO	ribosomal protein L15 [rpc operon]	148 ± 14	98.1	99.9	1	1	1	1	1	1	1	1	1	1	1	1	2
K02881	rplR	ribosomal protein L18 [rpc operon]	124 ± 19	99.3	99.9	1	1	1	1	1	1	1	1	1	1	1	2	2
K02890	rplV	ribosomal protein L22 [S10 operon]	123 ± 21	99.3	99.8	1	1	1	1	1	1	1	1	1	1	0	2	2
K02892	rplW	ribosomal protein L23 [S10 operon]	99 ± 11	97.3	99.7	1	1	1	1	1	1	1	1	1	1	0	1	2
K02895	rplX	ribosomal protein L24 [rpc operon]	106 ± 11	99.0	99.5	1	1	1	1	1	1	1	1	1	0	1	2	2
K02904	rpmC	ribosomal protein L29 [S10 operon]	69 ± 12	96.7	99.8	1	0	1	1	1	1	1	1	1	1	1	2	4
K02967	rpsB	ribosomal protein S2	260 ± 29	98.1	99.9	1	1	1	1	1	1	1	1	1	1	1	1	2
K02986	rpsD	ribosomal protein S4	203 ± 10	98.4	96.4	1	1	1	1	1	1	1	1	1	1	1	1	3
K02992	rpsG	ribosomal protein S7	159 ± 12	99.7	99.7	1	1	0	0	1	0	1	0	1	2	1	2	3
K02996	rpsI[d]	ribosomal protein S9	138 ± 15	98.7	99.9	1	1	1	1	1	1	1	1	1	1	1	2	3
K02946	rpsJ[d]	ribosomal protein S10 [S10 operon]	103 ± 4	97.6	99.7	1	1	1	1	1	1	1	1	1	1	1	3	1
K02950	rpsL	ribosomal protein S12	129 ± 11	97.7	99.6	1	1	0	0	1	0	0	0	1	2	1	2	3
K02956	rpsO	ribosomal protein S15	93 ± 16	97.7	99.8	1	2	1	1	1	1	1	1	1	1	1	2	4
K02961	rpsQ	ribosomal protein S17 [S10 operon]	90 ± 14	97.7	99.8	1	1	1	1	1	1	1	1	1	1	1	2	3
K02965	rpsS[d]	ribosomal protein S19 [S10 operon]	95 ± 15	97.3	99.8	1	1	1	1	1	0	1	1	1	1	1	2	2
K02926	rplD[d]	ribosomal protein L4 [S10 operon]	209 ± 17	98.8	99.7	1	1	1	1	1	1	1	1	1	1	0	1	3
K02935	rplL[d]	ribosomal protein L7/L12	125 ± 6	98.0	99.9	1	1	1	0	1	1	1	1	1	2	0	2	2
K02939	rplI	ribosomal protein L9	156 ± 22	97.0	99.9	1	1	1	1	1	1	1	1	1	1	0	1	3
K02878	rplP[d]	ribosomal protein L16 [S10 operon]	139 ± 7	98.7	99.8	1	1	1	1	1	1	1	1	1	1	0	2	7
K02879	rplQ	ribosomal protein L17 [alpha operon]	138 ± 31	97.7	99.8	1	1	1	1	1	1	1	1	1	1	0	2	3
K02884	rplS[d]	ribosomal protein L19	122 ± 13	97.6	99.8	1	1	1	2	1	1	1	1	1	0	0	2	4
K02887	rplT[d]	ribosomal protein L20	120 ± 9	98.7	99.9	1	1	1	1	1	1	1	1	1	1	0	2	2
K02888	rplU	ribosomal protein L21	109 ± 20	96.8	99.9	1	1	1	1	1	1	1	1	1	1	0	2	1
K02899	rpmA[d]	ribosomal protein L27	89 ± 7	97.6	99.9	1	1	1	1	1	1	1	1	1	1	0	2	1
K02916	rpmI	ribosomal protein L35	66 ± 5	97.0	99.9	1	1	1	1	1	1	1	0	1	1	0	2	1
K02990	rpsF	ribosomal protein S6	119 ± 23	97.1	100.0	1	1	1	1	1	2	1	1	1	1	0	1	1
K02959	rpsP	ribosomal protein S16	104 ± 33	99.0	99.8	1	1	1	2	1	1	1	1	1	0	0	1	2
K02968	rpsT	ribosomal protein S20	88 ± 8	97.8	99.9	1	1	1	1	1	1	1	1	1	1	0	2	2
K03040	rpoA	DNA-directed RNA polymerase, alpha subunit [alpha operon]	330 ± 16	98.4	98.5	1	1	1	1	1	1	1	1	1	1	0	2	3
K03110	ftsY	signal recognition particle-docking protein FtsY	392 ± 86	97.1	99.9	0	1	1	1	1	1	1	1	1	1	1	2	3
K01890	pheT	phenylalanyl-tRNA synthetase, beta subunit	797 ± 44	97.7	99.9	1	1	1	1	1	1	1	1	1	1	1	2	5
K03076	secY	preprotein translocase, SecY subunit [rpc operon]	441 ± 21	99.1	95.1	1	1	1	2[h]	1	1	1	1	1	1	1	1	3
K01868	thrS	threonyl-tRNA synthetase	634 ± 51	99.1	93.4	1	1	1	1	1	1	1	1	1	1	1	2	10
K02528	ksgA	dimethyladenosine transferase	279 ± 22	98.1	98.8	1	1	1	1	1	1	1	1	1	1	1	2	2
K01881	proS	prolyl-tRNA synthetase	543 ± 55	98.4	95.1	1	1	1	1	1	3	1	1	1	1	1	3	4
K01866	tyrS	tyrosyl-tRNA synthetase	412 ± 18	99.5	94.2	1	1	1	1	1	0	1	1	1	1	1	1	5

Supplementary TableS3. *Continued.*

Number of ORF annotated to specific KO within bin-genomes[g]: 0 | 1 | 2 | 3 | >4

KO	Genesymbol	Description	Average peptide length ± SD	House keeping (%)	Single copied (%)	Bin-genome: Bac1	Bac2	Bet1	Unc1	Unc2	DM1	DB1	DF1	DB2	DF2	MS1[i]	Chl1mix	DM2mix
K01870	ileS	isoleucyl-tRNA synthetase	983 ± 75	98.2	97.3	1	1	1	1	0	2[h]	1	1	1	1	1	2	10
K02601	nusG	transcription termination/antitermination factor NusG	193 ± 39	97.6	97.5	1	1	0	0	1	1	1	1	1	1	1	2	2
K06942	ychF	GTP-binding protein YchF	367 ± 10	99.1	99.6	1	1	0	1	1	1	1	1	2	3	1	2	6
K01887	argS	Arginyl-tRNA synthetase	571 ± 38	97.4	95.4	1	1	1	1	1	0	1	1	1	0	1	2	4
K03702	uvrB	excinuclease ABC, B subunit	687 ± 65	96.7	99.4	1	2	1	1	1	3[h]	1	1	1	0	1	2	4
K01876	aspS	aspartyl-tRNA synthetase	584 ± 28	98.5	98.8	1	1	1	1	1	0	1	0	0	1	1	2	6
K01872	alaS	Alanyl-tRNA synthetase	865 ± 80	98.3	99.1	1	1	1	2	0	4	1	0	1	1	2	1	3
K01892	hisS	histidyl-tRNA synthetase	434 ± 25	98.4	96.8	1	1	3	1	4	0	1	0	1	1	1	2	5
K01883	cysS	cysteinyl-tRNA synthetase	473 ± 39	97.3	97.3	1	1	2	1	1	2[h]	2	2	1	2	1	2	4
K02343	dnaX	DNA polymerase III, subunits gamma and tau	614 ± 141	97.5	95.3	1	1	1	1	4	0	2	3[h]	1	1	1	2	6
K01937	pyrG[d]	CTP synthase	544 ± 15	94.1	99.8	1	1	1	1	1	2	1	1	2	0	1	2	7
K00927	pgk[d]	phosphoglycerate kinase	399 ± 15	94.5	98.4	1	1	1	1	1	1	1	0	0	2	1	2	2
K02834	rfbA	ribosome-binding factor A	133 ± 47	97.0	99.9	1	1	1	1	1	1	1	1	1	1	0	2	5
K02838	frr[d]	ribosome recycling factor	185 ± 5	98.6	99.9	1	1	1	1	1	1	1	1	1	1	0	2	3
K02357	tsf[d]	translation elongation factor Ts	285 ± 36	98.1	99.9	1	1	1	1	1	1	1	1	1	1	0	1	4
K02835	prfA	peptide chain release factor 1	358 ± 12	97.9	99.8	1	1	1	1	1	3	1	1	1	1	0	3	4
K03977	engA	ribosome-associated GTPase EngA	467 ± 46	97.3	99.8	1	1	1	1	1	1	1	1	1	1	0	5	4
K04075	tilS	tRNA(Ile)-lysidine synthetase	413 ± 61	96.4	99.8	1	1	1	1	1	1	1	1	1	0	0	2	2
K02519	infB	translation initiation factor IF-2	865 ± 127	98.5	99.6	1	1	2	1	1	2[h]	2	1	3[h]	1	0	2	6
K03664	smpB[d]	SmpB protein	156 ± 10	98.0	99.5	1	1	1	1	1	1	1	1	1	0	0	1	2
K03596	lepA	GTP-binding protein LepA	604 ± 22	98.4	99.4	1	1	2	1	1	1	1	1	1	1	0	2	6
K03545	tig	trigger factor	445 ± 23	97.9	99.4	1	1	1	1	0	1	2	2[h]	1	1	0	2	2
K03438	mraW	MraW methylase family	318 ± 22	98.5	99.3	1	1	1	1	1	1	1	1	1	1	0	2	3
K03685	rnc	ribonuclease III	243 ± 42	96.0	99.0	1	1	1	1	0	1	1	1	1	1	0	1	6
K02520	infC[d]	translation initiation factor IF-3	176 ± 39	96.4	98.7	1	1	1	1	1	1	1	1	1	1	0	2	2
K03043	rpoB[d]	DNA-directed RNA polymerase, beta subunit	1274 ± 108	95.5	99.5	1	1	1	0	2	3[h]	1	1	1	2	0	2	8
K03046	rpoC	DNA-directed RNA polymerase, beta' or beta'' subunit	1335 ± 148	96.5	97.4	1	1	1	0	2	4	1	2	1	3	0	2	12
K02316	dnaG[d]	DNA primase	606 ± 57	97.8	98.2	0	4	1	1	2	1	1	2	1	1	0	2	2
K03553	recA	recA protein	358 ± 61	96.6	96.8	1	1	2	2	7	0	1	1	1	1	0	2	1
K02963	rpsR	ribosomal protein S18	83 ± 11	98.2	96.8	1	1	1	1	0	1	1	1	1	1	0	0	1
K00942	gmk	guanylate kinase	207 ± 24	95.7	96.6	1	1	1	1	1	1	1	1	1	1	0	2	6
K02338	dnaN	DNA polymerase III, beta subunit	375 ± 16	98.3	94.7	1	1	1	1	2	1	1	1	1	2	0	3	2
K02902	rpmB	ribosomal protein L28	75 ± 13	96.1	94.4	0	1	1	1	1	1	1	1	0	0	0	1	4
K02313	dnaA	chromosomal replication initiator protein DnaA	469 ± 54	96.6	94.1	2	1	1	1	1	1	1	1	1	1	0	3	4
K07042	ybeY	probable rRNA maturation factor	164 ± 31	93.8	99.9	1	1	1	1	1	1	1	1	0	1	0	2	1
K03075	secG	preprotein translocase, SecG subunit	105 ± 30	95.2	99.8	1	1	0	0	0	1	1	1	0	2	0	1	1
K00859	coaE	dephospho-CoA kinase	212 ± 42	94.9	99.7	1	1	0	1	0	0	1	1	1	1	0	3	2
K03073	secE	preprotein translocase, SecE subunit	92 ± 32	94.7	99.6	1	1	0	0	1	1	1	1	1	1	0	2	2
K03595	era	GTP-binding protein Era	306 ± 19	92.2	99.6	1	1	1	0	0	1	1	1	1	2	0	2	3
K04043	dnaK[f]	chaperone protein DnaK	629 ± 35	97.9	91.6	1	2	4	1	2	5	1	2	1	4	1	5	8
K02470	gyrB[f]	DNA gyrase, B subunit	724 ± 94	97.6	88.5	1	1	2	1	1	2	1	1	1	2	1	3	1
K01972	ligA[f]	DNA ligase, NAD-dependent	675 ± 80	96.9	88.3	1	2[h]	1	1	2[h]	1	1	1	1	1	0	2	5
K02469	gyrA[f]	DNA gyrase, A subunit	852 ± 82	97.6	86.6	0	1	1	1	2	2	1	1	1	1	1	4	8
K03070	secA[f]	preprotein translocase, SecA subunit	889 ± 79	99.2	86.1	1	1	1	3	1	2[h]	1	1	1	1	0	2	5
K02911	rpmF[f]	ribosomal protein L32	59 ± 7	93.5	96.6	0	0	1	0	1	1	0	1	0	0	0	2	1
K00566	mnmA[f]	tRNA (5-methylaminomethyl-2-thiouridylate)-methyltransferase	370 ± 25	95.0	93.7	1	1	2	1	1	3[h]	1	1	1	1	0	2	3
K02914	rpmH[f]	ribosomal protein L34	46 ± 4	88.2	99.9	0	0	0	1	0	0	0	0	0	0	0	1	2
K01879	glyS[f]	glycyl-tRNA synthetase beta chain	693 ± 32	64.6	100.0	0	0	2[h]	0	0	3	1	1	0	0	0	0	6
K01869	leuS[f]	leucyl-tRNA synthetase	859 ± 61	47.4	26.6	1	1	1	2	1	1	1	1	1	1	1	2	10

[a] Bacterial genomes having the housekeeping gene / Total 1943 KEGG organisms (in Nov, 2012) × 100 (%). Red colored, <95%; Orange colored, <97%.

[b] Bacterial genomes having the housekeeping gene with single copy / Total bacterial genomes having the housekeeping gene × 100 (%). Red colored, <95%; Orange colored, <97%.

[c] Sixteen single-copied housekeeping genes (eight for ribosomal proteins) used for core gene-based microbial community analysis.

[d] Thirty protein encoding phylogenetic marker genes used for AMPHORA program (Wu et al. 2008). The column yellow-highlighted.

[e] Three single-copied housekeeping genes (rplE, nusA, and pheS) used for phylogenetic tree analysis.

[f] Ten single-copied housekeeping genes were not used for draft genome completeness estimation because of their low qualities.

[g] Column was colored depend on number of ORF annotated to specific KO within each bin-genome. White column was counted for draft genome completeness.

[h] Column showed multi-copied partial ORFs annotated to same KO, and the sum of the peptide length was similar to averaged peptide length, which treated as single copy existence.

[i] Blue letter with orange highlighted column indicates genes that are not conserved in 133 *Archaea* genomes (in Nov, 2012).

Supplementary Table S4. Single copied gene list for validation of draft genome assembly within domain *Archaea* [a]

KO	KO discription	Bin-genome MS1	*Methanosalsum zhilinae*	*Methanococcoides burtonii*
K00096	glycerol-1-phosphate dehydrogenase [NAD(P)] [EC:1.1.1.261]	1	1	1
K00555	tRNA (guanine-N2-)-methyltransferase [EC:2.1.1.32]	1	1	1
K00586	diphthine synthase [EC:2.1.1.98]	1	1	1
K00609	aspartate carbamoyltransferase catalytic subunit [EC:2.1.3.2]	1	1	1
K00611	ornithine carbamoyltransferase [EC:2.1.3.3]	1	1	1
K00946	thiamine-monophosphate kinase [EC:2.7.4.16]	1	1	1
K01251	adenosylhomocysteinase [EC:3.3.1.1]	1	1	1
K01265	methionyl aminopeptidase [EC:3.4.11.18]	1	1	1
K01591	orotidine-5'-phosphate decarboxylase [EC:4.1.1.23]	1	1	1
K01803	triosephosphate isomerase (TIM) [EC:5.3.1.1]	1	1	1
K01807	ribose 5-phosphate isomerase A [EC:5.3.1.6]	1	1	1
K01868	threonyl-tRNA synthetase [EC:6.1.1.3]	1	1	1
K01870	isoleucyl-tRNA synthetase [EC:6.1.1.5]	1	1	1
K01872	alanyl-tRNA synthetase [EC:6.1.1.7]	2	1	1
K01873	valyl-tRNA synthetase [EC:6.1.1.9]	1	1	1
K01874	methionyl-tRNA synthetase [EC:6.1.1.10]	1	1	1
K01876	aspartyl-tRNA synthetase [EC:6.1.1.12]	1	1	1
K01880	glycyl-tRNA synthetase [EC:6.1.1.14]	1	1	1
K01881	prolyl-tRNA synthetase [EC:6.1.1.15]	1	1	1
K01885	glutamyl-tRNA synthetase [EC:6.1.1.17]	1	1	1
K01887	arginyl-tRNA synthetase [EC:6.1.1.19]	1	1	1
K01889	phenylalanyl-tRNA synthetase alpha chain [EC:6.1.1.20]	1	1	1
K01890	phenylalanyl-tRNA synthetase beta chain [EC:6.1.1.20]	1	1	1
K01892	histidyl-tRNA synthetase [EC:6.1.1.21]	1	1	1
K01937	CTP synthase [EC:6.3.4.2]	1	1	1
K01975	2'-5' RNA ligase [EC:6.5.1.-]	**0** [b]	1	1
K02117	V-type H+transporting ATPase subunit A [EC:3.6.3.14]	1	1	1
K02118	V-type H+transporting ATPase subunit B [EC:3.6.3.14]	1	1	1
K02528	16S rRNA -dimethyltransferase [EC:2.1.1.182]	1	1	1
K02601	transcriptional antiterminator NusG	1	1	1
K02683	DNA primase [EC:2.7.7.-]	1	1	1
K02863	large subunit ribosomal protein L1	1	1	1
K02864	large subunit ribosomal protein L10	1	1	1
K02866	large subunit ribosomal protein L10e	1	1	1
K02867	large subunit ribosomal protein L11	1	1	1
K02869	large subunit ribosomal protein L12	1	1	1
K02871	large subunit ribosomal protein L13	1	1	1
K02874	large subunit ribosomal protein L14	1	1	1
K02876	large subunit ribosomal protein L15	1	1	1
K02877	large subunit ribosomal protein L15e	1	1	1
K02881	large subunit ribosomal protein L18	1	1	1
K02883	large subunit ribosomal protein L18e	1	1	1
K02885	large subunit ribosomal protein L19e	1	1	1
K02886	large subunit ribosomal protein L2	1	1	1
K02889	large subunit ribosomal protein L21e	1	1	1
K02890	large subunit ribosomal protein L22	**0** [b]	1	1
K02895	large subunit ribosomal protein L24	1	1	1
K02896	large subunit ribosomal protein L24e	1	1	1
K02906	large subunit ribosomal protein L3	1	1	1
K02907	large subunit ribosomal protein L30	1	1	1
K02910	large subunit ribosomal protein L31e	1	1	1
K02912	large subunit ribosomal protein L32e	1	1	1
K02921	large subunit ribosomal protein L37Ae	1	1	1
K02927	large subunit ribosomal protein L40e	**0** [b]	1	1
K02929	large subunit ribosomal protein L44e	**0** [b]	1	1
K02930	large subunit ribosomal protein L4e	1	1	1
K02931	large subunit ribosomal protein L5	1	1	1
K02933	large subunit ribosomal protein L6	1	1	1
K02936	large subunit ribosomal protein L7Ae	1	1	1
K02946	small subunit ribosomal protein S10	1	1	1
K02948	small subunit ribosomal protein S11	1	1	1
K02950	small subunit ribosomal protein S12	1	1	1
K02952	small subunit ribosomal protein S13	1	**0**	1
K02956	small subunit ribosomal protein S15	1	1	1
K02961	small subunit ribosomal protein S17	1	1	1
K02965	small subunit ribosomal protein S19	1	1	1
K02966	small subunit ribosomal protein S19e	1	1	1
K02967	small subunit ribosomal protein S2	1	1	1
K02974	small subunit ribosomal protein S24e	1	1	1
K02978	small subunit ribosomal protein S27e	**0** [b]	1	1

Supplementary Table S4. *Continued.*

KO	KO discription	Bin-genome MS1	*Methanosalsum zhilinae*	*Methanococcoides burtonii*
K02979	small subunit ribosomal protein S28e	1	1	1
K02982	small subunit ribosomal protein S3	1	1	1
K02984	small subunit ribosomal protein S3Ae	1	1	1
K02986	small subunit ribosomal protein S4	1	1	1
K02987	small subunit ribosomal protein S4e	1	1	1
K02988	small subunit ribosomal protein S5	1	1	1
K02991	small subunit ribosomal protein S6e	1	1	1
K02992	small subunit ribosomal protein S7	1	1	1
K02994	small subunit ribosomal protein S8	1	1	1
K02995	small subunit ribosomal protein S8e	1	1	1
K02996	small subunit ribosomal protein S9	1	1	1
K03041	DNA-directed RNA polymerase subunit A' [EC:2.7.7.6]	1	1	1
K03042	DNA-directed RNA polymerase subunit A'' [EC:2.7.7.6]	1	1	1
K03047	DNA-directed RNA polymerase subunit D [EC:2.7.7.6]	1	1	1
K03049	DNA-directed RNA polymerase subunit E' [EC:2.7.7.6]	1	1	1
K03050	DNA-directed RNA polymerase subunit E'' [EC:2.7.7.6]	**0**[b]	1	1
K03053	DNA-directed RNA polymerase subunit H [EC:2.7.7.6]	1	1	1
K03055	DNA-directed RNA polymerase subunit K [EC:2.7.7.6]	**0**[b]	1	1
K03056	DNA-directed RNA polymerase subunit L [EC:2.7.7.6]	1	1	1
K03058	DNA-directed RNA polymerase subunit N [EC:2.7.7.6]	**0**[b]	1	1
K03076	preprotein translocase subunit SecY	1	1	1
K03106	signal recognition particle subunit SRP54	1	1	1
K03110	fused signal recognition particle receptor	1	1	1
K03232	elongation factor 1-beta	**0**[b]	1	**0**
K03234	elongation factor 2	1	1	1
K03237	translation initiation factor 2 subunit 1	1	1	1
K03242	translation initiation factor 2 subunit 3	1	1	1
K03243	translation initiation factor 5B	1	1	1
K03263	translation initiation factor 5A	1	1	1
K03264	translation initiation factor 6	1	1	1
K03265	peptide chain release factor subunit 1	1	1	1
K03330	glutamyl-tRNA(Gln) amidotransferase subunit E [EC:6.3.5.7]	1	1	1
K03470	ribonuclease HII [EC:3.1.26.4]	1	1	1
K03626	nascent polypeptide-associated complex subunit alpha	1	1	1
K03627	putative transcription factor	1	1	1
K04483	DNA repair protein RadA	1	1	1
K04794	peptidyl-tRNA hydrolase, PTH2 family [EC:3.1.1.29]	1	1	1
K04795	fibrillarin-like pre-rRNA processing protein	**0**[b]	1	1
K04798	prefoldin beta subunit	1	1	1
K04799	flap endonuclease-1 [EC:3.-.-.-]	1	1	1
K04800	replication factor C large subunit	1	1	1
K06174	ATP-binding cassette, sub-family E, member 1	1	1	1
K06865	ATPase	1	1	1
K06875	programmed cell death protein 5	1	1	1
K06943	nucleolar GTP-binding protein	**0**[b]	1	1
K06944	Predicted GTPase	**0**[b]	1	1
K06961	ribosomal RNA assembly protein	1	1	1
K06965	protein pelota	1	1	1
K07041	Predicted metal-dependent Rnase	1	1	1
K07060	UPF0271 protein	1	1	1
K07178	RIO kinase 1 [EC:2.7.11.1]	1	1	1
K07558	tRNA nucleotidyltransferase (CCA-adding enzyme) [EC:2.7.7.72]	1	1	1
K07561	diphthamide synthase subunit DPH2	1	1	1
K07566	tRNA threonylcarbamoyladenosine biosynthesis protein	1	1	1
K07572	putative nucleotide binding protein	1	1	1
K07575	PUA domain protein	1	**0**	1
K07580	hypothetical protein	**0**[b]	1	1
K07583	tRNA pseudouridine synthase 10 [EC:5.4.99.-]	2	1	1
K07732	riboflavin kinase, archaea type [EC:2.7.1.161]	1	1	1
K09141	hypothetical protein	1	1	1
K09482	glutamyl-tRNA(Gln) amidotransferase subunit D [EC:6.3.5.7]	1	1	1
K09735	hypothetical protein	1	1	1
K09903	uridylate kinase [EC:2.7.4.22]	1	1	1
K11130	H/ACA ribonucleoprotein complex subunit 3	**0**[b]	1	1
K14415	tRNA-splicing ligase RtcB [EC:6.5.1.3]	1	1	1
K14574	ribosome maturation protein SDO1	1	1	1

[a] Values indicate numbers of ORFs annotated to KO of Archaeal housekeeping genes.

[b] Orange column means missing gene to estimate draft genome completeness

Supplementary Table S5. Summary of Bin-genomes clustered from metagenomic assembly.

			HMP draft genome criteria[a] Criteria for pass	(1) >90%	(2) > 5.0	(3) >5 kb	(4) >20 kb	(5) >5 kb	(6) > 90%		
Bin-genome ID	Length	Contigs (=scaffold)[b]	Longest contig	% Contigs >500bp	Ave Contig Cov	Contig w/o N N50[b]	Contig (Scaffold) N50[b]	Ave Contig Length	% Complete-ness[c]	Meet Criteria[a]	Genome status
DB1	3,511,546	79	170,436	**100**	**81.3**	**61,819**	**87,111**	**44,450**	**95**	6/6	High-quality draft [HQD]
DB2	2,487,597	114	91,763	**100**	**23.0**	**23,717**	**40,483**	**21,821**	**93**	6/6	High-quality draft [HQD]
DF1	3,522,993	166	168,890	**100**	**29.7**	**22,242**	**49,879**	**21,223**	**90**	6/6	High-quality draft [HQD]
DF2	3,350,215	262	84,666	**100**	**22.5**	**15,453**	19,991	**12,787**	78	4/6	Standard draft [SD]
DM1	2,861,175	908	40,180	**100**	**25.6**	4,102	4,662	3,151	84	1/6	Standard draft [SD]
Bet1	2,589,546	487	41,557	**100**	**31.9**	**7,221**	8,781	**5,317**	87	3/6	Standard draft [SD]
Bac1	2,978,359	86	163,566	**100**	**37.8**	**51,788**	**73,194**	**34,632**	**96**	6/6	High-quality draft [HQD]
Bac2	4,592,223	218	196,280	**100**	**21.3**	**33,443**	**48,979**	**21,065**	**96**	6/6	High-quality draft [HQD]
Unc1	3,600,550	58	258,628	**100**	**31.5**	**44,269**	**111,785**	**62,078**	80	5/6	Standard draft [SD]
Unc2	2,534,126	73	280,490	**100**	**26.4**	**51,096**	**60,663**	**34,714**	82	5/6	Standard draft [SD]
MS1	2,212,198	171	61,973	**100**	**257.6**	**20,458**	**25,353**	**12,937**	**90**	6/6	High-quality draft [HQD]
DM2mix	7,729,897	3843	39,649	**100**	**13.5**	na	2,823	2,011	347[d]	2/5	Mixed[d]
Chl1mix	3,881,265	302	149,429	**100**	**24.8**	na	**20,405**	**12,852**	190[d]	4/5	Mixed[d]

[a] The draft genome quality was validated by using criteria described HMP Microbial Reference Genome work (The Human Microbiome Jumpstart Reference Strains Consortium, Science 328:994-999).

[b] In this study, contigs were scaffold with N by CLC de novo assembly cell. Thereby, for criteria (3), scaffolds were separated to contigs at N region.

[c] Values were calculated from frequency of KO assignment to universal single-copied gene family lists (Supplementary Table S4 and S5).

[d] Chl1mix was considered as mixture of two genomes, while DM2mix was considered as mixture of over three genomes.

Supplementary Table S6. Bin-genome linkage to highly abundant phylotypes (>1%) observed in 16S rRNA clone analysis.

16S clone analysis		Bin-genome linkage		16S in metagenome[e]						
Clone ID	No. of clone	Bin-genome ID[b]	Cov. range	Length of contig	Cov. of contig	Phylum (Class)	Family	% match	Closest strain	Accession no.
**Domain *Bacteria***										
Wcon1_M01	**8**	DF2	20-30	37503	21.9	*Deltaproteobacteria*	*Desulfobacteraceae*	99	*Desulfobacter postgatei* DSM 2034	AF418180
Wcon1_M03	**7**	DF1[a]	26-35	-	-	*Deltaproteobacteria*	*Desulfobacteraceae*	96	*Desulfococcus biacutus* DSM 5651	AJ277887
Wcon1_M09	**3**	-	-	7910	20.5	*Deltaproteobacteria*	*Desulfobacteraceae*	94	*Desulfonema magnum*	U45989
Wcon1_I05	**26**	DB1	70-90	44551	99.7	*Deltaproteobacteria*	*Desulfobulbaceae*	91	*Desulfobulbus rhabdoformis* Mic5c02	AB546248
Wcon1_C21	**11**	DB2	20-30	802	204.6	*Deltaproteobacteria*	*Desulfobulbaceae*	98	*Desulfobacterium catecholicum*	EF442982
Wcon2_A24	**6**	DM1[a]	20-50	-	-	*Deltaproteobacteria*	*Desulfuromonadaceae*	99	*Desulfuromonas acetexigens*	U23140
Wcon1_O21	**4**	DM2mix[a]	10-20	-	-	*Deltaproteobacteria*	*Geobacteraceae*	98	*Geobacter hephaestius*	AY737507
Wcon1_M21	**5**	-	-	-	-	*Deltaproteobacteria*	*Syntrophobacteraceae*	95	*Syntrophobacter wolinii*	U49429
Wcon1_K23	**10**	Bet1	23-50	1128	221.2	*Betaproteobacteria*	*Rhodocyclaceae*	96	*Denitratisoma oestradiolicum* AcBE2-1	NR_043249
Wcon1_I01	**3**	-	-	-	-	*Betaproteobacteria*	*Hydrogenophilaceae*	98	*Thiobacillus thioparus* THI 115	HM535225
Wcon1_G05	**5**	-	-	919	33.8	*Epsilonproteobacteria*	*Campylobacterales*	100	*Arcobacter cryaerophilus*	U34387
Wcon1_C05	**10**	-	-	-	-	*Clostridia*	*Peptostreptococcacea*	98	*Clostridium maritimum* G12	EU089965
Wcon1_G17	**3**	-	-	673	63.6	*Spirochaetes*	-	95	*Spirochaetes* bacterium SA-10	AY695841
Wcon2_K14	**1**	Bac2	18-26	23828	20.3	*Bacteroidetes*	-	91	*Bacteroidetes* bacterium 4F6B	AB623230
Wcon1_O17	**7**	Chl1mix[a]	20-32	-	-	*Chloroflexi*	*Anaerolineae*	97	Bacterium JN18_A7_F	DQ168648
Wcon1_O19	**6**	Unc1	28-40	66003	29.7	Unclassified	-	83	*Moorella thermoacetica* ATCC 39073	CP000232
Wcon1_K03	**5**	Chl1mix	20-32	16203	22.7	Unclassified	-	87	Bacterium Ellin 513	AY960776
Wcon1_G13	**3**	-	-	585	46.5	Unclassified	-	87	Candidatus Isobeggiatoa divolgata	FN561862
Wcon2_A06	**6**	-	-	4753	68.1	Unclassified	-	84	*Deltaproteobacterium* BABL1	GQ495224
Two-copy clones[c]	**28**					- 14 phylotypes observed				
Single clones[d]	**102**					- 103 phylotypes observed (except Wcon2_K14)				
SUM	**259**									
**Domain *Archaea***										
wMFCa_M03	**83**	MS1	200-500	8364	448.1	*Euryarchaeota*	*Methanosarcinaceae*	99	*Methanomethylovorans hollandica*	NR_102454
SUM	**83**									

[a] Bin-genome linkage was estimated by comparison of phylogenetic positions between 16S rRNA and three housekeeping proteins (NusA, PheS, and RplE) (Supplementary Fig. S2-S5).
[b] Bin-genome Bac1 and Unc2 were not linked to their detailed phylogenetic positions from these comparison analyses.
[c] Total 14 different phylotypes were observed with two clones in 259 clones.
[d] Total 103 different phylotypes were observed with single clones in 259 clones.
[e] JCVI MG-pipeline extracted 16S rRNA region from the assembled contigs. The length and average coverage of the contigs involving the 16S rRNA region were shown.

Supplementary Table S7. Summary of Bin-genomes clustered from metagenomic assembly.

Taxonomy of contigs Bin-genome ID	Core gene-basis frequency (%)[a]	Length (Mbp)	Number of contigs	Numbers of gene/ORF[b]	KO-assigned ORFs	Number of c-type cytochromes[c]	Raw reads mapped[d]	Raw reads frequency (%)[d]
Desulfobulbaceae	**0.1** ± 0.3	0.82	708	1338	634	18 (12)	121,266	0.2
Bin-genome DB1	**7.2** ± 0.7	3.51	79	3188	1607	92 (46)	3,105,519	6.2
Bin-genome DB2	**1.8** ± 0.3	2.49	114	2217	1281	24 (8)	603,250	1.2
Desulfobacteraceae	**4.0** ± 1.0	12.96	8496	19016	8831	132 (51)	1,864,146	3.7
Bin-genome DF1	**2.7** ± 0.5	3.52	166	3272	1710	37 (18)	1,186,533	2.4
Bin-genome DF2	**1.5** ± 0.5	3.35	262	2967	1611	27 (10)	807,030	1.6
Desulfuromonadales								
DM2mix	**2.9** ± 0.7	7.73	3843	10512	5427	138 (76)	1,206,183	2.4
Bin-genomeDM1	**2.4** ± 0.6	2.86	908	3361	1797	77 (54)	852,428	1.7
Syntrophobacterales	**3.4** ± 1.3	8.30	6483	12807	6957	73 (24)	1,290,512	2.6
Other *Deltaproteobac*	**1.4** ± 1.0	16.80	10460	24346	10301	234 (98)	2,852,956	5.7
Betaproteobacteria	**2.8** ± 0.9	9.41	6524	14993	6345	109 (26)	1,491,950	3.0
Bin-genome Bet1	**3.3** ± 0.5	2.59	487	3020	1771	62 (17)	1,051,762	2.1
Gammaproteobacteria	**0.3** ± 0.4	8.60	7081	13570	4588	81 (19)	1,319,582	2.6
Alphaproteobacteria	**0.3** ± 0.3	4.84	4325	7967	2949	26 (9)	634,864	1.3
Other Proteobacteria	**0.7** ± 0.9	6.47	1017	7555	2421	53 (16)	2,048,828	4.1
Bacteroidetes	**3.0** ± 1.0	17.03	13885	24523	10062	76 (17)	1,689,278	3.4
Bin-genome Bac1	**3.1** ± 0.3	2.98	86	2514	1278	14 (5)	1,199,373	2.4
Bin-genome Bac2	**1.7** ± 0.2	4.59	218	3748	1620	39 (11)	1,031,135	2.1
Chloroflexi	**0.6** ± 0.6	3.74	3533	6112	2801	18 (2)	447,061	0.9
Chl1mix	**3.9** ± 1.1	3.88	302	3715	1935	19 (1)	1,047,413	2.1
Firmicutes	**9.6** ± 2.8	21.08	16991	33792	16969	128 (19)	2,639,034	5.3
Phylum *Mixed*[e]	**5.3** ± 2.6	14.05	5216	17683	8025	95 (22)	2,719,207	5.5
Bin-genome Unc1	**2.7** ± 1.1	3.60	58	3081	1559	50 (26)	1,270,440	2.5
Bin-genome Unc2	**2.2** ± 0.9	2.53	73	2634	806	17 (0)	680,490	1.4
Euryarchaeota	**1.2** ± 2.3	7.71	5800	11094	4485	42 (5)	1,944,592	3.9
Bin-genome MS1	**22.7** ± 2.5	2.21	171	2155	1204	10 (2)	5,683,384	11.4
Other Phyla	**9.2** ± 1.9	70.78	72454	118711	27683	345 (120)	9,048,985	18.2
Total	100	248.5	169740	359891	136657	2036 (714)	49,837,201	100

[a] Averaged frequency within the metagenome was calculated based on the coverage of 13 universal single-copied core genes (Mean ± SD).

[b] Numbers of ORFs have potential errors in metagenomic ORF calling because of incomplete assemblies.

[c] Number of ORFs assigned as *c*- type cytochrome by CXXCH domain search. Numbers of multi-heme *c*- type cytochromes are descrined in parentheses.

[d] Raw reads frequency to the bin-genomes was calculated by read mapping of raw DNA reads to contigs.

[e] Contig was assigned to phylum "*Mixed*" when several phyla classified to ORFs within single contigs not met to contig taxnomic binning criteria.

Supplementary Table S8. Summary of mRNA read counts and mRNA/DNA ratio of bin-genomes.

Taxonomy of contigs Bin-genome ID	No. of gene/ORF[a]	No. of CDSs[b]	Normalized total reads mapped to ORFs[c]				mRNA/DNA ratio		
			DNA	MFC mRNA	SP mRNA	OC mRNA	MFC	SP	OC
Desulfobulbaceae	1338	349	13220	391	502	470	0.40	0.51	0.48
Bin-genome DB1	3188	2657	357161	6801	16537	12604	**0.25**	**0.62**	**0.47**
Bin-genome DB2	2217	1681	66988	5092	6731	7263	**1.02**	**1.34**	**1.45**
Desulfobacteraceae	19016	6954	200806	12507	11344	11181	0.83	0.76	0.74
Bin-genome DF1	3272	3042	131407	26412	26118	28771	2.69	2.66	2.93
Bin-genome DF2	2967	2463	90121	19472	19345	18272	2.89	2.87	2.71
Desulfuromonadales DM2mix	10512	4453	131946	7269	8533	10861	**0.74**	**0.87**	**1.10**
Bin-genomeDM1	3361	2352	96450	9265	11678	12489	**1.29**	**1.62**	**1.73**
Syntrophobacterales	12807	4826	137645	11063	10031	10314	1.08	0.98	1.00
Other *Deltaproteobacteria*	24346	6338	314757	14240	13319	14779	0.61	0.57	0.63
Betaproteobacteria	14993	2126	176664	1891	1496	1502	0.14	0.11	0.11
Bin-genome Bet1	3020	907	137938	1119	906	1099	0.11	0.09	0.11
Gammaproteobacteria	13570	2117	153189	2293	2027	2356	0.20	0.18	0.21
Alphaproteobacteria	7967	1058	72696	1249	1200	1161	0.23	0.22	0.21
Other Proteobacteria	7555	2117	235246	4518	3799	4554	0.26	0.22	0.26
Bacteroidetes	24523	2345	187970	1401	1304	1271	0.10	0.09	0.09
Bin-genome Bac1	2514	999	140756	971	830	949	0.09	0.08	0.09
Bin-genome Bac2	3748	879	120067	909	732	706	0.10	0.08	0.08
Chloroflexi	6112	1109	49687	1074	1095	1145	0.29	0.29	0.31
Chl1mix	3715	1427	122223	1621	1431	1571	0.18	0.16	0.17
Firmicutes	33792	4973	295457	4002	3650	3832	0.18	0.17	0.17
Phylum *Mixed*[d]	17683	2930	314723	3050	2709	2708	0.13	0.12	0.12
Bin-genome Unc1	3081	1345	149983	1782	1369	1598	0.16	0.12	0.14
Bin-genome Unc2	2634	255	83890	192	170	160	0.03	0.03	0.03
Euryarchaeota	11094	3043	221138	7956	6956	5913	0.48	0.42	0.36
Bin-genome MS1	2155	2071	660724	57989	51441	46861	1.17	1.04	0.95
Other Phyla	118711	13849	961865	12801	12077	12941	0.18	0.17	0.18
Total	359891	78665	5624719	217331	217331	217331	15.8	16.4	16.8

[a] Numbers of ORFs have potential errors in metagenomic ORF calling because of incomplete assemblies.

[b] Numbers of CDSs were counted of ORFs where at least one mRNA read was mapped on the criteria of 50% length cut-off and 95% identity.

[c] Raw reads counts to the bin-genomes wwere calculated by read mapping of raw DNA or mRNA reads to ORFs. The total DNA reads were calcurated as means of three operational conditions. The total mRNA reads of each condition were normalized by DNA reads of each condition.

[d] Contig was assigned to phylum "*Mixed*" when several phyla classified to ORFs within single contigs not met to contig taxnomic binning criteria.

Supplementary Table S9. Top10 highly-expressed annotated ORFs in Bin-genome DB1 under three operational conditions.

Peptide ID on JCVI metagenomic pipeline	Peptide Length	UniRef Common name	KO	CytC family[b]	MFC[a] mRNA (RPKM)	SP[a] mRNA (RPKM)	OC[a] mRNA (RPKM)	DNA[c] (RPKM)	Contig ID[d]	ORF Start[d]	ORF End[d]
**[condition MFC]**											
orf.1133802016390.1	136	-	-	-	2882.9	**473.5**	**116.7**	27.1	contig_833	66044	66452
orf.1133784620494.1	218	-	-	-	2039.9	**1318.9**	**7352.2**	22.8	contig_722	10882	11536
orf.1133802155256.1	237	prepilin-type cleavage/methylation N-terminal domain protein	-	-	1446.6	947.8	584.4	20.7	contig_138	86006	86717
orf.1133802016396.1	105	-	-	-	1422.5	**114.1**	**13.7**	26.9	contig_833	66494	66809
orf.1133802016560.1	399	cytochrome c family protein, putative	-	1964 (7)	1084.7	**93.8**	**10.8**	25.6	contig_833	67802	68999
orf.1133802088848.1	406	polysulfide reductase NrfD	-	-	1015.9	**520.1**	**1083.8**	21.8	contig_1236	11359	12577
orf.1133803259248.1	814	putative flagellin protein	K02406	-	952.9	723.0	1058.1	23.8	contig_879	123802	126244
orf.1133802642656.1	153	putative hspC2 heat shock protein	K13993	-	898.6	685.2	471.5	26.0	contig_58	16821	17280
orf.1133802016578.1	207	cytochrome B561	-	-	869.1	**79.6**	**20.9**	15.4	contig_833	69064	69685
orf.1133784620364.1	161	-	-	-	780.1	**474.4**	**6155.9**	22.9	contig_722	10066	10549
orf.1133802022530.1	341	PEP-CTERM putative exosortase interaction domain	-	-	691.9	579.7	**165.0**	26.5	contig_96	20041	21064
orf.1133802804278.1	91	-	-	-	559.5	411.4	428.0	19.6	contig_359	55164	55437
orf.1133802293742.1	458	-	-	-	548.5	654.0	570.1	25.2	contig_771	388	1762
orf.1133802088804.1	90	-	-	-	528.0	**266.2**	**593.1**	24.2	contig_1236	10924	11194
orf.1133803319958.1	92	histone family protein DNA-binding protein	K03530	-	498.1	732.5	**407.7**	25.1	contig_3709	13259	13535
orf.1133803055150.1	223	peptidoglycan-associated lipoprotein	K03640	-	426.2	**1074.5**	802.2	17.0	contig_2301	3386	4055
orf.1133802016588.1	251	-	-	- (1)	385.4	**11.9**	11.5	13.9	contig_833	69414	70167
orf.1133784620342.1	103	-	-	-	362.5	276.2	**3445.6**	19.4	contig_722	9580	9889
orf.1133802088862.1	132	transcriptional regulator, merR family	K13640	-	360.0	**56.7**	**196.7**	24.3	contig_1236	12645	13041
**[additional genes in condition SP]**											
orf.1133803564556.1	55	-	-	-	0.0	**6861.5**	**1678.7**	24.9	contig_948	55708	55873
orf.1133803564564.1	71	-	-	-	0.0	**6475.3**	**3352.6**	21.7	contig_948	55885	56098
orf.1133784552376.1	81	-	-	-	230.5	**6082.7**	**2511.3**	23.1	contig_90	23140	23383
orf.1133803564578.1	193	-	-	-	70.4	**5260.8**	**2982.5**	20.4	contig_948	56155	56734
orf.1133784552372.1	203	-	-	-	16.7	**4249.2**	**1186.8**	20.0	contig_90	23436	24045
orf.1133784552518.1	459	-	K07090	-	55.5	**1742.3**	1452.1	21.6	contig_90	21732	23109
orf.1133784552542.1	293	uspA domain protein	-	-	17.4	**1466.9**	1654.4	21.6	contig_90	20882	21761
orf.1133803540748.1	66	-	-	-	128.6	**703.4**	**240.4**	34.9	contig_345	100580	100778
orf.1133803564446.1	551	chaperonin groL	K04077	-	73.9	**521.8**	**149.2**	20.7	contig_948	9500	11153
orf.1133803564400.1	145	chaperonin 10 Kd subunit	K04078	-	35.1	**464.8**	**79.6**	19.0	contig_948	11252	11687
**[additional genes in condition OC]**											
orf.1133784620352.1	71	-	-	-	310.8	**147.6**	**2194.5**	18.6	contig_722	9944	10157
orf.1133802523796.1	108	-	-	-	172.9	**97.1**	**1389.2**	24.7	contig_259	65724	66048
orf.1133802522876.1	401	-	K02004	-	42.3	37.3	**528.9**	18.2	contig_259	66060	67263
orf.1133801860664.1	128	ribosomal protein L7/L12	K02935	-	0.0	**58.5**	**439.6**	22.3	contig_2562	3358	3742
orf.1133801860656.1	176	ribosomal protein L10	K02864	-	9.6	**110.6**	**434.4**	23.1	contig_2562	2791	3319
orf.1133802110998.1	104	ribosomal protein S10	K02946	-	32.6	**144.0**	**402.3**	23.6	contig_12942	41	353

[a] Highly-expressed genes were picked from biggest mRNA RPKM to tenth UniRef annotated gene for each condition (yellow highlighted).
Upregulated genes showed red letters, while down-regulated genes showed blue letters.

[b] *c*- type cytochrome family was defined by Bulter *et al.* (BMC Genomics, 2010, 11:40). Number of heme binding CXXCH domain is described in paraphrase.

[c] DNA frequency (DNA-RPKM) was an average of three operational conditions.

[d] Potential operons (gene cluster within the contig) were highlighted by different colors.

Supplementary Table S10. Top10 highly-expressed annotated ORFs in Bin-genome DM1 under three operational conditions.

Peptide ID on JCVI metagenomic pipeline	Peptide Length	UniRef Common name	KO	CytC family[b]	MFC[a] mRNA (RPKM)	SP[a] mRNA (RPKM)	OC[a] mRNA (RPKM)	DNA[c] (RPKM)	Contig ID[d]	ORF Start[d]	ORF End[d]
**[condition MFC]**											
orf.1133794964200.1	303	citrate (Si)-synthase[e]	K01647	-	4883.9	**1254.6**	1767.5	5.6	contig_81654	0	908
orf.1133793756148.1	102	citrate (Si)-synthase[e]	K01647	-	1936.6	**441.9**	**879.8**	9.3	contig_203048	203	508
orf.1133785393793.1	98	class III cytochrome C family	-	2890 (3)	1610.7	1176.7	1177.7	8.8	contig_41276	299	593
orf.1133802795474.1	537	acetyl-coA hydrolase/transferase	-	-	1093.6	**237.0**	**486.3**	6.8	contig_2418	7803	9414
orf.1133803043328.1	277	-	-	-	913.0	654.2	656.2	10.0	contig_4502	5644	6475
orf.1133802117908.1	49	lipoprotein	-	-	796.7	**489.0**	441.6	18.5	contig_12609	849	996
orf.1133801731582.1	833	formate C-acetyltransferase	K00656	-	768.2	715.5	583.6	9.5	contig_10352	3207	5706
orf.1133795138440.1	319	malate dehydrogenase, NAD-dependent	K00024	-	713.0	591.5	587.9	8.7	contig_65882	282	1239
orf.1133803529874.1	79	-	-	-	687.5	**227.5**	**1059.2**	14.7	contig_800	6497	6734
orf.1133796818988.1	97	-	-	-	682.4	**262.5**	**937.0**	6.9	contig_44061	2633	2924
orf.1133803043304.1	135	-	-	-	678.9	**321.7**	299.2	6.0	contig_4502	2641	3046
orf.1133785521143.1	76	-	-	-	580.7	**236.5**	**835.2**	14.7	contig_53727	1	229
orf.1133802310598.1	518	NADH-quinone oxidoreductase, chain M	K00342	-	560.3	**286.2**	370.4	5.1	contig_8254	2914	4468
orf.1133803043292.1	248	succinate dehydrogenase subunit B	K00240	-	554.4	410.6	383.9	7.5	contig_4502	3109	3853
orf.1133803529886.1	90	-	-	-	528.0	332.8	**897.6**	12.2	contig_800	6232	6502
orf.1133803043276.1	639	succinate dehydrogenase, flavoprotein subunit	K00239	-	499.4	339.8	438.0	7.2	contig_4502	3849	5766
orf.1133802310514.1	168	NADH-ubiquinone/plastoquinone oxidoreductase, chain 6	K00339	-	495.0	**240.7**	352.1	5.7	contig_8254	2	506
orf.1133801927240.1	330	-	-	64omcS (3)	473.2	322.2	271.0	6.0	contig_10083	8233	9223
**[additional genes in condition SP]**			-								
orf.1133803459982.1	89	ATP synthase F0, C subunit	K02110	-	152.6	**1295.6**	891.5	8.0	contig_6703	3115	3382
orf.1133803545608.1	118	acyl carrier protein	K02078	-	244.5	**888.4**	978.1	12.8	contig_134	2572	2926
orf.1133795138470.1	199	2-oxoglutarate ferredoxin oxidoreductase, alpha subunit	K00174	-	427.2	**851.8**	755.2	6.1	contig_65882	1619	2215
orf.1133801962216.1	181	ribosomal protein L10	K02864	-	112.5	**843.9**	765.2	9.7	contig_14006	602	1145
orf.1133802318530.1	143	ribosomal protein L20	K02887	-	189.9	**795.9**	696.1	10.1	contig_5810	9094	9523
orf.1133802644130.1	86	ribosomal protein L22	K02890	-	138.2	**783.6**	469.7	7.3	contig_7413	18625	18883
orf.1133795138452.1	64	iron-sulfur cluster-binding protein	K00176	-	397.8	**702.0**	698.8	6.2	contig_65882	1428	1620
**[additional genes in condition OC]**											
orf.1133802347582.1	177	ribosomal protein L21	K02888	-	191.8	**499.2**	627.6	11.1	contig_7819	1765	2296

[a] Highly-expressed genes were picked from biggest mRNA RPKM to tenth UniRef annotated gene for each condition (yellow highlighted). Upregulated genes showed red letters, while down-regulated genes showed blue letters.

[b] *c*- type cytochrome family was defined by Bulter *et al.* (BMC Genomics, 2010, 11:40). Number of heme binding CXXCH domain is described in paraphrase.

[c] DNA frequency (DNA-RPKM) was an average of three operational conditions.

[d] Potential operons (gene cluster within the contig) were highlighted by different colors.

[e] These two citrate synthases were probably misconnected. Those genes were counted as a single gene for picking tem most expressed genes.

Supplementary Table S11. Top10 highly-expressed annotated ORFs in Bin-genomes DF1, DF2, and DB2 under three operational conditions.

Peptide ID on JCVI metagenomic pipeline	Peptide Length	UniRef Common name	KO	CytC family	MFC[a] mRNA (RPKM)	SP[a] mRNA (RPKM)	OC[a] mRNA (RPKM)	DNA[b] (RPKM)	Contig ID[c]	ORF Start[c]	ORF End[c]
Bin-genome DF1											
**[condition MFC]**											
orf.1133803263250.1	46	-	-	-	2376.2	1763.8	1955.6	15.4	contig_2329	0	136
orf.1133802880730.1	502	adenylylsulfate reductase, alpha subunit	K00394	-	2180.8	2303.0	2566.3	8.0	contig_3549	424	1930
orf.1133802880684.1	67	adenosine-5'-phosphosulfate reductase alpha subunit	K00394	-	2102.6	2034.0	2174.7	5.9	contig_3549	0	201
orf.1133794978062.1	105	aprA (fragment)	K00394	-	1718.9	2174.8	2274.2	8.4	contig_80176	388	702
orf.1133802867116.1	438	-	-	-	1677.9	1795.0	2150.8	7.3	contig_1743	9147	10461
orf.1133802700666.1	438	sulfite reductase, dissimilatory-type alpha subunit	K11180	-	1658.5	1504.4	1788.5	8.5	contig_10831	19743	21057
orf.1133802700806.1	383	sulfite reductase, dissimilatory-type beta subunit	K11181	-	1639.7	1454.5	1909.7	8.0	contig_10831	18579	19728
orf.1133803003758.1	120	-	-	-	1555.8	**873.6**	**1887.5**	6.7	contig_892	167749	168109
orf.1133802169864.1	94	ATP synthase F0, C subunit	K02110	-	1282.0	1258.6	1841.7	9.5	contig_3114	31355	31637
orf.1133802700820.1	100	dissimilatory sulfite reductase D	-	-	1273.0	1198.0	865.6	10.0	contig_10831	18267	18567
orf.1133784536440.1	391	adh4	K13954	-	1224.1	**693.2**	752.7	8.5	contig_10504	4459	5632
orf.1133802831506.1	423	sulfate adenylyltransferase	K00958	-	1143.6	1210.8	1244.8	9.4	contig_7483	17933	19202
orf.1133801811484.1	62	4Fe-4S binding domain	-	-	1067.6	**1739.1**	1303.0	8.0	contig_1245	9715	9901
**[additional genes in condition SP]**											
orf.1133802813022.1	87	-	-	-	897.4	**1824.6**	**3100.9**	7.2	contig_3060	46367	46628
orf.1133803041966.1	675	V-type H(+)-translocating pyrophosphatase	K15987	-	990.7	1109.3	1258.8	9.1	contig_296	6571	8596
**[additional genes in condition OC]**											
orf.1133802813032.1	94		-	-	433.3	**701.0**	**1473.3**	7.9	contig_3060	46059	46341
orf.1133784607782.1	106	sulfur relay protein, tusE/dsrC/dsvC family	K11179	-	960.7	1045.5	1088.8	7.4	contig_970	24102	24420
Bin-genome DF2											
**[condition MFC]**											
orf.1133785657059.1	155	-	-	-	2452.9	1942.0	1805.6	5.7	contig_51019	5143	5608
orf.1133785656869.1	127	adenylylsulfate reductase, beta subunit	K00395	-	1977.9	1922.1	1806.2	4.8	contig_51019	5662	6043
orf.1133802313416.1	401	translation elongation factor Tu	K02358	-	1794.6	1385.5	1259.2	4.8	contig_2574	2178	3381
orf.1133797985438.1	68	-	K03704	-	1672.3	2290.4	2142.8	3.8	contig_20813	22756	22960
orf.1133802580362.1	384	sulfite reductase, dissimilatory-type beta subunit	K11181	-	1644.2	1255.8	1115.8	6.8	contig_3719	5149	6301
orf.1133794482838.1	332	porC1	K00174	-	1421.2	1204.4	1216.7	7.0	contig_61326	1711	2707
orf.1133803395114.1	269	aprA	K00394	-	1381.8	1224.8	1099.4	4.5	contig_6732	0	807
orf.1133802073614.1	215	succinate dehydrogenase cytochrome B subunit, b558 family	K00241	-	1349.9	1448.8	1207.8	5.0	contig_9969	10534	11179
orf.1133802353762.1	516	acetyl-coA hydrolase	K01067	-	1325.6	1099.9	752.1	5.6	contig_14426	12939	14487
orf.1133802073670.1	638	succinate dehydrogenase, flavoprotein subunit	K00239	-	1258.3	1030.4	1035.6	5.4	contig_9969	11207	13121
orf.1133802580324.1	442	sulfite reductase, dissimilatory-type alpha subunit	K11180	-	1178.9	1016.4	943.3	5.8	contig_3719	6313	7639
orf.1133794482804.1	61	porC1	K00174	-	1168.6	810.2	1158.9	3.9	contig_61326	1337	1520
orf.1133802073690.1	264	4Fe-4S ferredoxin iron-sulfur binding domain protein	K00240	-	1144.4	896.3	857.9	5.5	contig_9969	13096	13888
orf.1133784586864.1	410	ATP citrate lyase subunit 1	K01648	-	1084.6	799.9	714.3	6.2	contig_10548	6122	7352
**[additional genes in condition SP]**											
orf.1133785657075.1	32	-	-	-	901.7	1170.0	**676.2**	3.7	contig_51019	5996	6092
orf.1133793461756.1	198	peptidoglycan-associated lipoprotein	K03640	-	857.2	1096.7	772.3	6.5	contig_96379	107	701
orf.1133794482802.1	70	fdx9	K00176	-	921.4	1091.1	845.0	4.8	contig_61326	1127	1337
**[additional genes in condition OC]**											
orf.1133802097708.1	70	ribosomal protein S21	K02970	-	388.0	**855.7**	968.6	6.8	contig_10218	14393	14603
orf.1133794482850.1	274	porB1	K00175	-	1015.9	869.0	874.0	6.4	contig_61326	2708	3530
Bin-genome DB2											
**[condition MFC]**											
orf.1133803592912.1	386	sulfite reductase, dissimilatory-type beta subunit	K11181	-	756.3	822.5	755.0	5.0	contig_3727	33125	34283
orf.1133786532312.1	141	adenylylsulfate reductase, beta subunit	K00395	-	650.0	743.5	552.5	7.1	contig_22118	11571	11994
orf.1133803592876.1	436	sulfite reductase, dissimilatory-type alpha subunit	K11180	-	603.4	642.3	602.2	6.2	contig_3727	34309	35617
orf.1133786532358.1	674	adenylylsulfate reductase, alpha subunit	K00394	-	599.3	631.0	590.8	4.7	contig_22118	12031	14053
orf.1133784579314.1	195	related to peptidoglycan-associated lipoprotein	K03640	-	530.9	529.9	562.3	5.6	contig_3883	1190	1775
orf.1133803220876.1	431	-	-	-	500.1	472.5	468.6	4.7	contig_4092	49820	51113
orf.1133803028242.1	112	sulfur relay protein, tusE/dsrC/dsvC family	K11179	-	500.1	427.9	502.4	8.4	contig_7987	1934	2270
orf.1133803323896.1	428	sulfate adenylyltransferase	K00958	-	460.0	405.9	337.1	6.5	contig_219	40329	41613
orf.1133803592922.1	86	probable dsrD protein	-	-	453.9	539.8	**268.4**	5.7	contig_3727	32781	33039
orf.1133803556266.1	404	probable alcohol dehydrogenase	K13954	-	382.3	**893.3**	1260.5	7.3	contig_3779	4795	6007
orf.1133803593538.1	99	ATP synthase F0, C subunit	K02110	-	325.7	272.3	320.6	6.0	contig_3727	13659	13956
**[additional genes in condition SP]**											
orf.1133802964310.1	398	translation elongation factor Tu	K02358	-	324.1	**662.2**	576.3	10.5	contig_1998	54593	55787
**[additional genes in condition OC]**											
orf.1133802964430.1	227	ribosomal protein S3	K02982	-	67.3	**270.5**	362.3	5.7	contig_1998	50279	50960
orf.1133802964020.1	128	ribosomal protein L7/L12	K02935	-	132.6	**245.7**	338.1	6.0	contig_1998	67826	68210

[a] Highly-expressed genes were picked from biggest mRNA RPKM to tenth UniRef annotated gene for each conditions for each conditions (yellow highlighted). Upregulated genes showed red letters, while down-regulated genes showed blue letters.

[b] DNA frequency (DNA-RPKM) was an average of three operational conditions.

[c] Potential operons (gene cluster within the contig) were highlighted by different colors.

Supplementary Table S12. Top10 highly-expressed annotated ORFs in Bin-genomes Bac1 and Bac2.

Peptide ID on JCVI metagenomic pipeline	Peptide Length	UniRef Common name	KO	CytC family	MFC[a] mRNA (RPKM)	SP[a] mRNA (RPKM)	OC[a] mRNA (RPKM)	DNA[b] (RPKM)	Contig ID[c]	ORF Start[c]	ORF End[c]
Bin-genome Bac1											
**[condition MFC]**											
orf.1133784698604.1	96	DNA-binding protein HU	K03530	-	106.1	31.2	30.1	10.4	contig_1046	61667	61955
orf.1133802754454.1	285	-	K03561	-	71.5	63.1	30.4	8.4	contig_9	135988	136843
orf.1133803606216.1	402	translation elongation factor Tu	K02358	-	59.1	37.3	39.5	9.6	contig_2880	14073	15279
orf.1133802847186.1	251	-	-	-	40.6	17.9	17.2	10.0	contig_5563	43998	44751
orf.1133802754486.1	222	-	-	-	38.2	33.7	26.0	10.0	contig_9	134641	135307
orf.1133803385764.1	139	domain of unknown function (DUF1987)	-	-	36.6	0.0	0.0	8.7	contig_2000	32691	33108
orf.1133784696812.1	93	ribosomal protein S19	K02965	-	36.5	48.3	0.0	14.4	contig_1046	29676	29955
orf.1133803606256.1	237	ribosomal protein L1	K02863	-	35.8	0.0	36.5	7.8	contig_2880	16724	17435
orf.1133785663937.1	95	histone family protein DNA-binding protein	K03530	-	35.7	0.0	0.0	5.8	contig_57761	36	321
orf.1133784696674.1	97	ribosomal protein L23	K02892	-	35.0	15.4	29.7	11.8	contig_1046	28558	28849
orf.1133803034752.1	153	small heat shock protein	K13993	-	33.3	58.7	56.6	9.9	contig_994	31539	31998
orf.1133803387704.1	315	fatty acid synthesis protein	K03621	-	32.3	9.5	0.0	11.1	contig_3689	4725	5670
orf.1133802754470.1	213	biopolymer transport protein exbD/tolR	-	-	31.9	7.0	40.6	9.7	contig_9	135303	135942
**[additional genes in condition SP]**											
orf.1133801837486.1	69	ribosomal protein L35	K02916	-	0.0	43.4	0.0	12.3	contig_10564	13361	13568
orf.1133784625080.1	71	-	-	-	23.9	42.2	0.0	11.9	contig_5625	78185	78398
orf.1133803491136.1	152	ribosomal protein L13	K02871	-	11.2	39.4	19.0	13.6	contig_69	18512	18968
orf.1133803349344.1	118	ribosomal protein S6	K02990	-	14.4	38.1	48.9	10.3	contig_382	48225	48579
orf.1133784487352.1	200	lipoprotein	-	-	17.0	37.4	72.1	11.0	contig_15141	288	888
orf.1133803606224.1	80	preprotein translocase, secE subunit	K03073	-	0.0	37.4	0.0	7.2	contig_2880	15386	15626
orf.1133802475648.1	123	-	-	-	0.0	36.5	23.5	12.0	contig_14733	11487	11856
orf.1133803606266.1	178	ribosomal protein L10	K02864	-	28.6	33.7	16.2	11.2	contig_2880	17457	17991
orf.1133784697016.1	90	ribosomal protein S14p/S29e	K02954	-	0.0	33.3	0.0	7.2	contig_1046	33415	33685
**[additional genes in condition OC]**											
orf.1133802367100.1	100	-	-	-	17.1	0.0	102.0	31.9	contig_1256	0	299
orf.1133784696630.1	102	ribosomal protein S10	K02946	-	0.0	0.0	56.6	13.3	contig_1046	26975	27281
orf.1133784698260.1	186	-	-	-	9.1	0.0	54.3	10.0	contig_1046	51286	51844
orf.1133784697006.1	189	50S ribosomal protein L5	K02931	-	0.0	0.0	53.4	9.3	contig_1046	32836	33403
orf.1133803606244.1	148	ribosomal protein L11	K02867	-	11.5	30.4	48.7	8.2	contig_2880	16277	16721
orf.1133784697074.1	61	ribosomal protein L30	K02907	-	0.0	0.0	47.3	8.7	contig_1046	35598	35781
orf.1133802754852.1	122	anti-anti-sigma factor	K04749	-	0.0	0.0	47.3	10.9	contig_9	23804	24170
orf.1133802847806.1	123	ribosomal protein L19	K02884	-	13.8	12.2	46.9	9.1	contig_5563	19510	19879
orf.1133803035064.1	93	ribosomal protein L31	K02909	-	0.0	0.0	46.5	11.5	contig_994	43099	43378
Bin-genome Bac2											
**[condition MFC]**											
orf.1133803304892.1	91	DNA-binding protein HU	K03530	-	111.9	32.9	63.4	5.0	contig_6307	27914	28187
orf.1133803309864.1	207	-	-	-	98.4	137.5	195.1	6.1	contig_9883	21204	21825
orf.1133801914020.1	157	-	-	-	75.7	57.2	18.4	4.7	contig_7536	19396	19867
orf.1133803595642.1	1332	-	-	-	73.9	56.2	40.1	17.8	contig_4930	79775	83771
orf.1133802498748.1	65	cold shock protein	K03704	-	52.2	0.0	0.0	5.0	contig_822	148970	149165
orf.1133785622539.1	1697	GLUG domain protein	-	-	50.0	23.8	21.3	5.4	contig_51489	0	5091
orf.1133791172874.1	36	-	-	-	47.1	41.6	0.0	45.7	contig_123242	843	951
orf.1133802066950.1	76	-	-	-	44.7	19.7	0.0	3.4	contig_4911	44320	44548
orf.1133802150312.1	648	NADH dehydrogenase (quinone)	K00335	-	44.5	76.3	55.7	5.4	contig_2111	69467	71411
orf.1133786266630.1	489	-	-	-	41.7	55.2	41.4	5.5	contig_31161	0	1466
orf.1133803476222.1	334	rhodanese-related sulfurtransferase	-	-	40.7	13.5	8.6	5.1	contig_3505	21148	22150
orf.1133802780984.1	214	-	-	-	39.7	42.0	6.7	5.6	contig_9142	22598	23240
orf.1133801988702.1	134	-	-	-	38.0	11.2	10.8	5.6	contig_12211	36181	36583
orf.1133802150046.1	187	response regulator receiver domain	K02488	-	36.3	8.0	15.4	6.1	contig_2111	71972	72533
orf.1133802499158.1	188	ribosomal protein L6	K02933	-	36.1	8.0	7.7	5.0	contig_822	12807	13371
orf.1133802022226.1	189	-	-	-	35.9	23.8	22.9	4.7	contig_16929	30912	31479
orf.1133784577104.1	97	DNA-binding protein HU	K03530	-	35.0	46.3	0.0	6.5	contig_7100	10287	10578
orf.1133797921988.1	396	translation elongation factor Tu	K02358	-	34.3	18.9	14.6	5.2	contig_32724	31771	32959
orf.1133784675958.1	450	glutamate dehydrogenase, NAD-specific	K00262	-	33.9	53.2	35.3	5.2	contig_12463	10076	11426
**[additional genes in condition SP]**											
orf.1133786266578.1	147	-	-	-	23.1	30.6	0.0	8.9	contig_31161	1708	2149
orf.1133803364410.1	98	ribosomal protein L31	K02909	-	0.0	30.6	29.4	3.6	contig_1450	62763	63057
orf.1133801977942.1	49	-	-	-	0.0	30.6	0.0	4.0	contig_15310	28572	28719
orf.1133802498670.1	156	ribosomal protein S7	K02992	-	10.9	28.8	9.2	2.8	contig_822	22620	23088
orf.1133802150062.1	166	NADH-quinone oxidoreductase, E subunit	K00334	-	10.2	27.1	26.1	6.5	contig_2111	71451	71949
orf.1133802499104.1	114	ribosomal protein L24	K02895	-	14.9	26.3	12.7	3.6	contig_822	14630	14972
orf.1133801807896.1	126	-	-	-	0.0	23.8	0.0	5.1	contig_11662	4082	4460
orf.1133802984176.1	132	ribosomal protein S9/S16	K02996	-	0.0	22.7	10.9	5.9	contig_139	50219	50615
orf.1133802150344.1	581	hydrogenase, Fe-only	K00336		14.6	20.6	9.9	5.0	contig_2111	67715	69458
**[additional genes in condition OC]**											
orf.1133797922122.1	146	ribosomal protein L11	K02867	-	23.3	10.3	29.6	6.1	contig_32724	30331	30769
orf.1133802498794.1	100	ribosomal protein L23	K02892	-	0.0	0.0	28.9	5.4	contig_822	18566	18866
orf.1133797922164.1	151	ribosomal protein L7/L12	K02935	-	22.5	0.0	28.7	6.4	contig_32724	28602	29055
orf.1133801922342.1	165	ATP synthase F0, B subunit	K02109	-	0.0	0.0	26.2	5.6	contig_3658	74733	75228
orf.1133797922140.1	232	ribosomal protein L1	K02863	-	0.0	6.5	24.9	4.9	contig_32724	29585	30281

[a] Highly-expressed genes were picked from biggest mRNA RPKM to tenth UniRef annotated gene for each conditions for each conditions (yellow highlighted). Upregulated genes showed red letters, while down-regulated genes showed blue letters.

[b] DNA frequency (DNA-RPKM) was an average of three operational conditions.

[c] Potential operons (gene cluster within the contig) were highlighted by different colors. To complete contig 2111 cluster, one hydrogenase gene was added.

Supplementary Table S13. Top10 highly-expressed annotated ORFs in Bin-genomes MS1 and Bet2.

Peptide ID on JCVI metagenomic pipeline	Peptide Length	UniRef Common name	KO	CytC family[b]	MFC[a] mRNA (RPKM)	SP[a] mRNA (RPKM)	OC[a] mRNA (RPKM)	DNA[c] (RPKM)	Contig ID[d]	ORF Start[d]	ORF End[d]
Bin-genome MS1											
**[condition MFC]**											
orf.1133802906078.1	385	S-layer-related duplication domain	-	-	8737.7	8689.7	9259.2	95.3	contig_282	13234	14389
orf.1133802591016.1	202	monomethylamine methyltransferase mtmB	-	-	8133.5	8192.1	7791.7	142.7	contig_3696	11429	12035
orf.1133802591038.1	252	monomethylamine methyltransferase mtmB	-	-	5590.3	6126.9	5993.9	134.8	contig_3696	12050	12806
orf.1133802590892.1	217	monomethylamine corrinoid protein 1	K00548	-	5209.2	5251.8	4773.4	132.9	contig_3696	10766	11417
orf.1133803173416.1	112	dimethylamine methyltransferase mtbB3	-	-	4864.5	3583.4	4276.4	128.7	contig_3272	2060	2396
orf.1133803173382.1	224	dimethylamine corrinoid protein 2	K00548	-	4372.0	3737.2	4070.3	132.4	contig_3272	3472	4144
orf.1133803173410.1	355	dimethylamine:corrinoid methyltransferase	-	-	4207.4	3020.4	3637.1	126.4	contig_3272	2396	3461
orf.1133802838476.1	254	methyltransferase cognate corrinoid proteins	K00548	-	4203.1	3348.9	3157.9	65.4	contig_1540	7333	8095
orf.1133802838514.1	467	methanol:corrinoid methyltransferase	-	-	3903.4	2655.2	2872.9	66.2	contig_1540	5927	7328
orf.1133802630752.1	434	methyl-coenzyme M reductase, beta subunit	K00401	-	3285.1	3322.9	3330.7	64.1	contig_790	11913	13215
orf.1133802630774.1	162	methyl-coenzyme M reductase operon protein D	K03422	-	2860.2	2662.3	2778.4	60.9	contig_790	13257	13743
orf.1133803063686.1	76	-	-	-	2858.6	4078.9	4309.0	52.7	contig_2308	20466	20694
orf.1133803173454.1	490	amino acid/polyamine transporter I	-	-	2539.0	1946.8	2293.5	57.9	contig_3272	483	1953
orf.1133802630956.1	572	methyl-coenzyme M reductase, alpha subunit	K00399	-	2513.3	2283.0	2428.8	64.5	contig_790	15125	16841
**[additional genes in conditions SP/OC]**											
orf.1133802630902.1	252	methyl-coenzyme M reductase, gamma subunit	K00402	-	2290.0	2347.4	2324.3	58.6	contig_790	14355	15111

Peptide ID on JCVI metagenomic pipeline	Peptide Length	UniRef Common name	KO	CytC family[b]	MFC[a] mRNA (RPKM)	SP[a] mRNA (RPKM)	OC[a] mRNA (RPKM)	DNA[c] (RPKM)	Contig ID[d]	ORF Start[d]	ORF End[d]
Bin-genome Bet1											
**[condition MFC]**											
orf.1133797721834.1	187	phasin family protein		-	453.8	352.4	200.6	10.6	contig_24274	894	1455
orf.1133802765784.1	493	gram-negative porin		-	216.9	230.9	184.4	12.6	contig_1537	35739	37218
orf.1133801963992.1	106	bacterial DNA-binding protein	K03530	-	176.1	**56.5**	68.0	8.5	contig_10282	160	478
orf.1133802530242.1	834	von willebrand factor type A		-	126.2	113.2	124.6	10.3	contig_7681	0	2501
orf.1133803112048.1	103	-	K03704	-	98.9	87.2	**28.0**	12.9	contig_1057	486	795
orf.1133802766018.1	165	putative type 4 fimbrial biogenesis transmembrane protein	K02655	-	82.3	**36.3**	61.2	9.6	contig_1537	13833	14328
orf.1133802773962.1	66	ribosomal protein L35	K02916	-	77.1	**0.0**	43.7	13.2	contig_3660	9092	9290
orf.1133803176920.1	92	ribosomal protein L7/L12	K02935	-	73.8	**16.3**	0.0	7.1	contig_1345	8606	8882
orf.1133803421980.1	107	-	-	-	64.0	70.6	81.7	11.7	contig_663	0	320
orf.1133802214864.1	251	cytochrome c4	-	- (2)	60.9	**23.9**	**5.7**	12.1	contig_11750	7827	8580
orf.1133803378028.1	672	adenylylsulfate reductase, alpha subunit	K00394	-	58.1	57.9	38.6	11.4	contig_9649	3023	5039
orf.1133802939964.1	121	-	-	-	56.1	49.5	35.8	7.4	contig_163	10528	10891
orf.1133802118342.1	215	-	-	-	55.3	62.7	67.1	11.1	contig_8029	3325	3970
orf.1133803580354.1	63	ribosomal protein L30	K02907	-	53.9	**23.8**	**0.0**	9.6	contig_2855	5134	5323
**[additional genes in condition SP]**											
orf.1133784456482.1	60	-	-	-	0.0	**49.9**	0.0	11.3	contig_8894	3927	4107
orf.1133803094172.1	418	membrane protein involved in aromatic hydrocarbon degradation	K06076	-	36.5	46.6	44.9	9.0	contig_75	19393	20647
orf.1133802144036.1	398	translation elongation factor Tu	K02358	-	34.1	45.2	29.0	29.8	contig_8037	5052	6246
orf.1133803070042.1	102	-	-	-	0.0	44.0	0.0	12.1	contig_7775	8071	8377
orf.1133797879228.1	69	-	-	-	0.0	43.4	0.0	3.7	contig_32915	2249	2456
orf.1133797869568.1	314	GDP-mannose 4,6-dehydratase	K01711	-	16.2	43.0	23.0	2.9	contig_29607	0	941
orf.1133802985956.1	70	-	-	-	24.2	42.8	0.0	11.0	contig_3162	9680	9890
orf.1133802606544.1	109	putative cytochrome c-552 nirM	-	- (1)	31.1	41.2	0.0	10.6	contig_2422	801	1128
orf.1133793146694.1	264	translation elongation factor Tu	K02358	-	12.9	39.7	5.5	8.9	contig_98833	0	792
**[additional genes in condition OC]**											
orf.1133802184272.1	196	peroxiredoxin	K03386	-	8.7	0.0	**110.4**	9.6	contig_2780	3426	4014
orf.1133803008226.1	99	-	-	-	17.1	0.0	58.3	6.6	contig_2955	3833	4130
orf.1133803243438.1	58	-	-	-	0.0	0.0	49.7	15.0	contig_358	3314	3488
orf.1133797879220.1	125	-	-	-	13.6	12.0	46.2	7.8	contig_32915	1855	2230
orf.1133786276180.1	98	adenylylsulfate reductase, beta subunit	K00395	-	0.0	30.9	44.6	14.6	contig_30872	1870	2163
orf.1133797776814.1	134	lytic transglycosylase catalytic	-	-	0.0	0.0	43.2	8.2	contig_21811	0	401

[a] Highly-expressed genes were picked from biggest mRNA RPKM to tenth UniRef annotated gene for each conditions for each conditions (yellow highlighted). Upregulated genes showed red letters, while down-regulated genes showed blue letters.

[b] *c*-type cytochrome family was defined by Bulter *et al.* (BMC Genomics, 2010, 11:40). Number of heme binding CXXCH domain is described in paraphrase.

[c] DNA frequency (DNA-RPKM) was an average of three operational conditions.

[d] Potential operons (gene cluster within the contig) were highlighted by different colors.

SUPPLEMENTARY DISCUSSION

Characterization of thirteen bin-genomes

To accomplish the recovery of bin-genomes of highly abundant microbes in the complex microbial community, we clustered the assembled contigs by using mean contig coverage vs. G+C content plots as reported elsewhere (1). The contig clusters were further analyzed by a set of criteria that considered the predicted taxonomy, mean coverage, G+C content, and contig length (Supplementary Tables S2).

Using this method, we recovered thirteen bin-genomes overall (Table 1). Six of these bin-genomes (MS1, DB1, DB2, DF1, Bac1, and Bac2) showed notably high estimated genome completeness based on observed coverage of single-copy housekeeping genes (Supplementary Table S3-S4) (2, 3), indicating nearly-complete draft genomes of these six strains were recovered from the metagenome. Bin-genomes DM1, DF2, and Bet1 showed relatively larger numbers of contigs and less complete genomes than the other bin-genomes. The lower quality of these specific bin-genomes is likely due to the presence of phylogenetically closely related strains that assembled with a similar mean coverage, and/or micro-diversity between the strains resulting in different mean coverage but notably similar genomes (4). However, the assembly quality for those bin-genomes was sufficient for the subsequent analyses.

On the other hand, two bin-genomes (Chl1mix and DM2mix) showed duplicate or triplicate existence of the noted single-copy genes (Supplementary Table S3), suggesting that those clusters were mixtures of genomes from at least two different microbes, and were omitted from further metabolic network analyses. In total, eleven bin-genomes were recovered from the new metagenomic assembly and determined to be the dominant strains within the community. These bin-genomes were then utilized for reconstructing the metabolic networks within the community by performing comparative analyses of their gene expression profiles between before and after stimuli addition.

The 16S rRNA gene is generally used to affiliate bin-genomes to their taxonomic position and estimate their potential role in the community; however, it is difficult to assemble highly conserved genomic regions such as 16S rRNA (Supplementary Table S5). Therefore, we used three housekeeping proteins (RplE, NusA, and PheS) for analyzing the phylogenetic positions and taxonomic classification of each bin-genome if the bin-genomes did not include 16S rRNA sequence information (Supplementary Fig. S3-S6). This method provided the taxonomic assignment of the eleven bin-genomes (Table 1) and led to the first estimation of functional roles for each strain; *i.e.*, family *Methanosarcinaceae* (MS1) as a methanogen (5), families *Desulfobacteraceae* (DF1 and DF2) and *Desulfobulbaceae* (DB1 and DB2) as dissimilatory sulfate reducers (6), and *Desulfuromonadaceae* (DM1) as a dissimilatory iron reducer (7). These results also indicate that we successfully identified a bin-genome of the most dominant *Desulfuromonadaceae* strain DM1 from *Desulfuromonadales* pan-genome DM of the previous study (1), which is important for analyzing the strain-based metabolic network.

Accurate community composition for complex microbial community

Conventional community analyses, i.e. evaluating community structure based on the relative abundance of small subunit rRNA gene of each taxon, is convenient. However, there are some unavoidable issues due to the inconsistent copy numbers of ribosomal RNA gene operon per genome (8), ununiformed compatibility of universal primers to the 16S rRNA genes from various organisms for the PCR amplification (9), and difficulty to design primers that can amplify both Archaeal and Bacterial 16S rRNA genes (10). Although the 16S rRNA clone analysis showed appropriate identification of phylotypes with phylogenetic positions (Supplementary Fig. S3), the community composition is incorrect due to the reasons described above (1).

To gain more accurate view of the community composition by overcoming the issues, the metagenomics-based community composition analysis has been examined because the next generation sequencing (NGS) is theoretically less-biased with no PCR amplification than 16S rRNA-basis sequencing. Taxonomic assignment of each ORF by similarity search against UniRef (11) or NCBI-NR (12) are frequently used for raw read or ORF frequencies-basis community composition analysis such as MEGAN (13) and MG-RAST (14). However, the results from this method are considerably affected by the accumulation of adequate reference genomes, and never address uncultured taxa with no reference genome and also phylotype level information of the given communities.

Since our bin-genome effort by contig clustering enables to show the strain-level information as well as the unclassified taxa (like bin-genomes Unc1 and Unc2) in the community (Table 1), raw reads frequencies for each bin-genome are another option to identify community composition (Supplementary Table S5). The raw reads frequencies related to bin-genome and taxonomic assignment of contigs showed relatively accurate view of the community composition; however, the approach has potential errors in terms of microbial cell numbers mainly attributed by size of (bin)genomes and raw reads contamination of virus/plasmid. The sizes of genomes directly affect to raw reads frequencies such as bigger genome size overestimates the frequency because of more raw reads existence. We found several contigs taxonomically assigned as Superkingdom *Viruses* within our metagenomic assembly. Those contigs seem to represent miscellaneous viruses infected and amplified in the microbial cell body. At the case, it is better to omit viruses-associated raw reads for microbial community composition analysis. The plasmid-associated contigs are similar contaminant to consider accurate community composition analysis because their copy numbers within the microbial cell body are highly variable.

To overcome all concerns described above, we used core-gene frequencies based community composition by relative frequencies of sixteen single-copied housekeeping genes for each bin-genome (Fig. 2). The core-gene frequency based community composition analysis along with metagenomics was first reported using 31 proteins as the phylogenetic markers for *Bacterial* population analyses (15), and using 105 proteins for Archaeal phylogenetic markers (16). However, in anaerobic microbial ecosystems, both *Bacteria* and *Archaea* are often existed together in a community; thereby, we selected core genes that are present in both domains' genomes for the community analyses (Fig. 2A). To the end, core-gene frequencies based community composition would be most suitable way to reflect microbial cell numbers composition within the given communities (Fig. 2C).

Stimulus-induced metatranscriptomics for natural environmental community

In the natural environments, microbial communities are sometimes highly diverse (17) because the selective pressures are low and variable (18). In those cases, metagenomic sequence data can be complex and analytically challenging (19) to obtain sufficient genome-binning; however, if dominant organisms occupy more than 1.5% of a given community, their bin-genomes will be associated using our level of metagenomic sequencing (~100M reads and ~10 Gbp). Given advances in sequencing technologies which can now provide high coverage sequence data, the recovery of draft genomes from the highly diverse samples will continue to improve.

In addition, natural environments generally include multiple variables that impact microbial activities. For example, a diurnal cycle and depth dependency includes light intensity as well as temperature and oxygen concentration differences (20-22), which are both significant environmental changes relative to microbial activity. If these two environmental factors are divided into individual stimuli (e.g. temperature or light), we can separately analyze the effect of each factor that controls individual microbial activity and metabolism, or interspecies interaction in the complex microbial communities. Since the microbial community should respond by changing gene expression relative to the specified applied stimulus (e.g. only light intensity), our proposed stimulus-induced metatranscriptomics approach with genome-binning should be suitable in many natural environments for further understanding how environmental perturbations impact microbial function at a strain- and community-level.

Supplementary Methods

Selection of marker genes for analyzing cell activity and microbial metabolisms

Microbial cell activity-associated marker genes were selected from the KEGG module or KEGG pathway databases (23). Transcription-associated marker gene sets are related to M00183 (RNA polymerase, bacteria), M00184 (RNA polymerase, Archaea), and bacterial sigma factors and other transcription-related factors from ko03021 (Transcription Machinery). Translation-associated marker gene sets are related to M00178 (Ribosome, bacteria), M00179 (Ribosome, Archaea), M00360 (prokaryotic Aminoacyl-tRNA biosynthesis), and M00335 (Sec system). Replication-associated marker gene sets are related to M00260 (Bacterial DNA polymerase III complex), M00264 (Archaeal DNA polymerase III complex), and *fts* and *dna* genes from ko03036 (Chromosome and associated proteins). Energy production-associated marker gene sets are related to M00157 (F-type ATPase) and M00159 (V-type ATPase), while oxidative stress response-associated genes are related to catalase or superoxide dismutase in KEGG orthology, and M00456 (anoxic redox control two-component regulatory system).

Microbial metabolism-associated marker genes were also selected from the KEGG module or KEGG pathway databases (23). Respiration-associated marker gene sets for soluble electron acceptors are related to M00567 (hydrogenotrophic methanogenesis), M00596 (Dissimilatory sulfate reduction), M00530 (Dissimilatory nitrate reduction), and ko00190 (Oxidative phosphorylation). EET respiration-associated marker gene sets are related to MH-cytC orthologous families established by Bulter *et al.* (24). The twelve key MH-cytC families were selected as cytochrome C_3 (PpcA, PpcD, PpcE, and family 2890), OmcS, OmcX, OmcB, OmcZ, OmcE, MacA, CymA, and family 51 (orf2 of cytochrome BC operon). Central carbon metabolism-associated marker genes are related to M00009/M00011 (Citrate cycle, TCA cycle), M00173 (Reductive citric acid cycle), M00001 (Glycolysis EMP), and M00003 (Gluconeogenesis). Substrate or byproduct-associated marker gene sets are related to M00377 (Reductive acetyl-CoA pathway), M00087 (beta-Oxidation), M00236 and M00237 (Amino acid transport systems), and M00239 (Peptides/Nickel transport system). The gene sets related to downstream process of acetyl-CoA are selected from ko00620 (Pyruvate metabolism), ko00010 (Glycolysis / Gluconeogenesis), and ko00640 (Propanoate metabolism). Hydrogenase gene sets are mainly selected from ko01000 (Enzymes) as EC:1.12.- (Oxidoreductase, Acting on hydrogen as donor) and manual selection of *hox* and *hyf* hydrogenase complex. Anaerobic sulfur metabolism-associated gene sets are selected from ko00920 (Sulfur metabolism) and *Desulfocapsa sulfexigens* metabolism (25).

SUPPLEMENTARY REFERENCES

1. Ishii S, *et al.* (2013) A novel metatranscriptomic approach to identify gene expression dynamics during extracellular electron transfer. *Nat Commun* 4:1601.
2. Dupont CL, *et al.* (2012) Genomic insights to SAR86, an abundant and uncultivated marine bacterial lineage. *ISME J* 6:1186-1199.
3. Kawai M, *et al.* (2011) Evolution in an oncogenic bacterial species with extreme genome plasticity: Helicobacter pylori East Asian genomes. *BMC Microbiol* 11:104.
4. Albertsen M, *et al.* (2013) Genome sequences of rare, uncultured bacteria obtained by differential coverage binning of multiple metagenomes. *Nature Biotech* 31:533-538.
5. Garcia JL, Patel BK, & Ollivier B (2000) Taxonomic, phylogenetic, and ecological diversity of methanogenic Archaea. *Anaerobe* 6(4):205-226.
6. Klein M, *et al.* (2001) Multiple lateral transfers of dissimilatory sulfite reductase genes between major lineages of sulfate-reducing prokaryotes. *J Bacteriol* 183:6028-6035.
7. Lovley DR, Holmes DE, & Nevin KP (2004) Dissimilatory Fe(III) and Mn(IV) reduction. *Adv Microb Physiol* 49:219-286.
8. Klappenbach JA, Saxman PR, Cole JR, & Schmidt TM (2001) rrndb: the Ribosomal RNA Operon Copy Number Database. *Nuc Acid Res* 29:181-184.
9. Sipos R, *et al.* (2007) Effect of primer mismatch, annealing temperature and PCR cycle number on 16S rRNA gene-targetting bacterial community analysis. *FEMS Microbiol Ecol* 60:341-350.
10. Baker GC, Smith JJ, & Cowan DA (2003) Review and re-analysis of domain-specific 16S primers. *J Microbiol Method* 55:541-555.
11. Suzek BE, Huang H, McGarvey P, Mazumder R, & Wu CH (2007) UniRef: comprehensive and non-redundant UniProt reference clusters. *Bioinformatics* 23:1282-1288.
12. Benson DA, *et al.* (2013) GenBank. *Nuc Acid Res* 41:D36-42.
13. Huson DH, Auch AF, Qi J, & Schuster SC (2007) MEGAN analysis of metagenomic data. *Genome Res* 17:377-386.
14. Meyer F, *et al.* (2008) The metagenomics RAST server - a public resource for the automatic phylogenetic and functional analysis of metagenomes. *BMC Bioinformatics* 9:386.
15. Wu M & Eisen JA (2008) A simple, fast, and accurate method of phylogenomic inference. *Genome Biol* 9:R151.
16. Wu M & Scott AJ (2012) Phylogenomic analysis of bacterial and archaeal sequences with AMPHORA2. *Bioinformatics* 28:1033-1034.
17. Nacke H, *et al.* (2011) Pyrosequencing-based assessment of bacterial community structure along different management types in German forest and grassland soils. *PloS One* 6:e17000.
18. Fierer N & Jackson RB (2006) The diversity and biogeography of soil bacterial communities. *PNAS* 103:626-631.
19. Delmont TO, *et al.* (2012) Structure, fluctuation and magnitude of a natural grassland soil metagenome. *ISME J* 6:1677-1687.
20. Kolber ZS, *et al.* (2001) Contribution of aerobic photoheterotrophic bacteria to the carbon cycle in the ocean. *Science* 292:2492-2495.
21. Poretsky RS, *et al.* (2009) Comparative day/night metatranscriptomic analysis of microbial communities in the North Pacific subtropical gyre. *Environ Microbiol* 11:1358-1375.

22. Klatt CG, *et al.* (2013) Temporal metatranscriptomic patterning in phototrophic Chloroflexi inhabiting a microbial mat in a geothermal spring. *ISME J* 7:1775-1789.

23. Kanehisa M, *et al.* (2008) KEGG for linking genomes to life and the environment. *Nucleic acids research* 36(Database issue):D480-484.

24. Butler JE, Young ND, & Lovley DR (2010) Evolution of electron transfer out of the cell: comparative genomics of six Geobacter genomes. *BMC Genomics* 11:40.

25. Finster KW, *et al.* (2013) Complete genome sequence of Desulfocapsa sulfexigens, a marine deltaproteobacterium specialized in disproportionating inorganic sulfur compounds. *Standards in genomic sciences* 8(1):58-68.
